# Novel 2-substituted-5-(4-chloro-2-phenoxy)phenyl-1,3,4-oxadiazole derivatives, ligands of GABAA/benzodiazepine receptor complex: Design, synthesis, radioligand binding assay, and pharmacological evaluation

**DOI:** 10.17179/excli2022-5639

**Published:** 2023-02-20

**Authors:** Elham Rezaee, Fatemeh Ahmadi, Mahsa Shabaninia, Mona Khoramjouy, Zahra Azizi Farsani, Soraya Shahhosseini, Sayyed Abbas Tabatabai, Mehrdad Faizi

**Affiliations:** 1 Department of Pharmaceutical Chemistry, School of Pharmacy, Shahid Beheshti University of Medical Sciences, Tehran, Iran; 2 Premier Care Long Term Care Pharmacy, North Little Rock, Arkansas, USA; 3 Phytochemistry Research Center, Shahid Beheshti University of Medical Sciences, Tehran, Iran; 4 Department of Pharmacology and Toxicology, School of Pharmacy, Shahid Beheshti University of Medical Sciences, Tehran, Iran

**Keywords:** benzodiazepine, binding assay, 1,3,4-oxadiazole, docking, hypnotic, anticonvulsant

## Abstract

Agonists of Benzodiazepine (BZD) receptor are exhaustively used in the control of muscle spasms, seizure, anxiety, and insomnia. BZDs have some unwanted effects; therefore, the development of new BZD receptor agonists with better efficacy and fewer unwanted effects is one of the subjects of interest. In this study, based on the pharmacophore/receptor model of the BZD binding site of GABA_A_ receptors, a series of new 2-substituted-5-(4-chloro-2-phenoxy)phenyl-1,3,4-oxadiazole derivatives (**6a**-**f**) were designed. Energy minima conformers of the designed compounds and diazepam were well matched in conformational analysis and showed proper interaction with the BZD-binding site of the GABA_A_ receptor model (α1β2ϒ2) in docking studies. The designed compounds were synthesized in acceptable yield and evaluated for their *in vitro* affinity to the benzodiazepine receptor of rat brains by radioligand receptor binding assay. The results demonstrated that the affinities of most of the novel compounds were even higher than diazepam. The novel compound **6a** with the best affinity in radioligand receptor binding assay (K_i_=0.44 nM and IC_50_= 0.73±0.17 nM) had considerable hypnotic activity and weak anticonvulsant and anxiolytic effects with no negative effect on memory in animal models. Flumazenil as a selective benzodiazepine receptor antagonist was able to prevent hypnotic and anticonvulsant effects of **6a** indicating the role of BZD receptors in these effects.

## Introduction

Gamma amino butyric acid (GABA) is the most significant inhibitory neurotransmitter in CNS and interacts with GABA_A_, GABA_B_, and GABA_C_ receptors. GABA_A_ receptor is a heterooligomeric complex that has seven different subunits (α1-6, β1-4, γ1-4, δ, ε, θ, and ρ1-3). The majority of GABA_A_ receptor subtypes contain α-, β- and γ-subunits arranged in a 2:2:1 stoichiometry and linked together as γβαβα, counter-clock-wise from synaptic cleft (Guerrini et al., 2008[10]). Many ligands, such as benzodiazepines (BZDs), barbiturates, ethanol, picrotoxin, and loreclezole can allosterically alter the effects of GABA on the GABA_A_ receptors (Carling et al., 2006[5]). Interaction of BZDs with the GABA_A_ receptor causes a conformational change in the receptor and the interface of α and γ subunits of the receptor is the main binding site (Guerrini et al., 2008[10]). This influences the binding properties of other ligands on the receptor and modulates the interaction of GABA on the chloride ion channel (Zarghi et al., 2008[20]). GABA_A_ receptor modulating drugs are common medicines on the market. These drugs act quickly, low toxicity, and pharmacological properties such as anxiolytic, anticonvulsant, muscle relaxant, and sedative-hypnotic effects (Lager et al., 2008[12]). Since BZDs have some unwanted reactions (sedation, negative effect on cognition, fatigue, ataxia, lethargy, and dependency) (Rivas et al., 2009[16]), many investigators are searching for novel BZD receptor agonists with different chemical classes to find novel ligands with less undesirable effects. 

**Figure 1:**
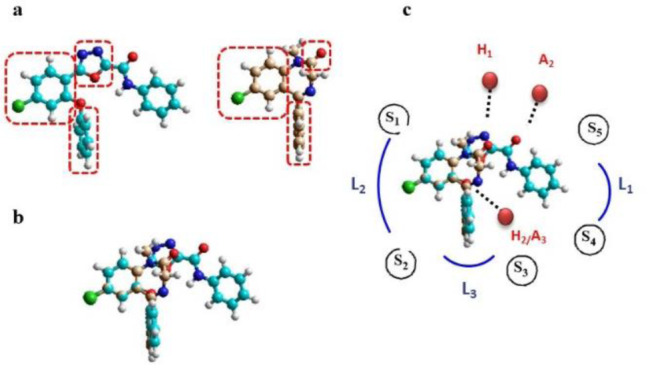
a: The structures of the designed compound (6a) and diazepam. The main pharmacophores have been conserved. b: Stereo view of the superimposition of the energy minima conformers of diazepam and compound 6a. c: Superimposition of diazepam and compound 6a located in the model suggested by Cook and co-workers (Zhang et al., 1995).

In a well-accepted model proposed for binding of ligands to the BZD receptors, an aromatic ring and a coplanar proton-accepting group, which is located at an appropriate distance from the ring, are necessary. Besides, an additional out-of-plane aromatic ring intensifies the affinity of the ligands to the receptor (Akbarzadeh et al., 2003[2]). Based on these features and to continue our previous studies on the compounds with five-membered heterocyclic core (Akbarzadeh et al., 2003[2]; Zarghi et al., 2005[18][21], 2008[19]; Faizi et al., 2012[8], 2015[6], 2017[7]; Almasirad et al., 2007[3]; Foroumadi et al., 2007[9]; Tabatabai et al., 2013[17]), the novel 1,3,4-oxadiazole derivatives (**6a-f**) with all the recommended necessities for binding to the BZD receptors were designed and synthesized (Figure 1a(Fig. 1)). We performed conformational analysis and docking studies and radioligand binding assay to check the affinity of the novel compounds to the BZD-binding site of GABA_A_ receptors. We selected the most potent compound and evaluated the anticonvulsant, hypnotic, and anxiolytic activity of the selected compound. We also evaluated the effect of the selected compound on memory using experimental models. Finally, flumazenil was used as a BZD receptor antagonist to prove the mode of action of the synthesized compounds.

## Results and Discussion

### Chemistry

**Figure 2:**
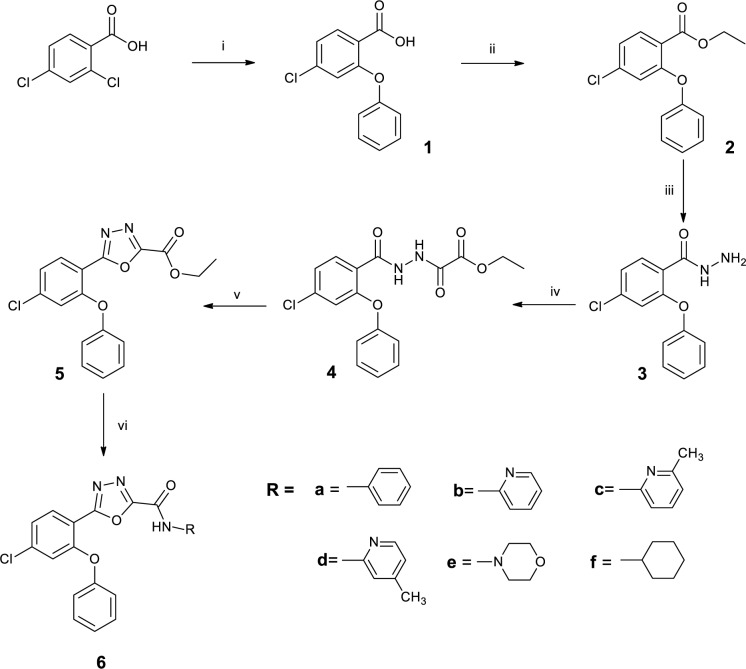
Reagents and conditions: i) phenol, potassium tert-butoxide, Cu, DMF, reflux, 5 h; ii) H_2_SO_4_, EtOH, reflux, 24 h; iii) NH_2_NH_2_.H_2_O, EtOH, rt, 12 h; iv) ethylchloroglyoxylate, triethylamine, THF, 0°C, 3 h; v) P_2_O_5_, toluene, reflux, 3 h; vi) proper amine, AlCl_3_, DCM, rt, 3-24h.

The designed novel compounds were synthesized according to Figure 2(Fig. 2). 4-chloro-2-phenoxybenzoic acid was prepared by the reaction of 2,4-dichlorobenzoic acid with phenol at 115-120 °C through an aromatic nucleophilic substitution reaction in acceptable yield (Faizi et al., 2012[8]). Compound **1** was converted to ethyl 4-chloro-2-phenoxybenzoate (**2**). Then, it reacted with hydrazine hydrate to afford the corresponding hydrazide **3**), which was treated with ethylchloroglyoxalate (Faizi et al., 2012[8]). Following the reaction of the intermediate (**4**) with P_2_O_5_ in toluene, the 1,3,4-oxadiazole ring was closed (Rezaee Zavareh et al., 2014[14]). Finally, six new amide derivatives were readily synthesized by the reaction of the ester (**5**) with proper amine (Bon et al., 1994[4]).

### Radioligand receptor binding assay

As shown in Table 1(Tab. 1), most of the novel compounds had a high affinity for BZD receptors compared to diazepam, a classic benzodiazepine receptor agonist. The most potent compound was **6a,** which had a phenyl substituent as its R group (Figure 2(Fig. 2) and Table 1(Tab. 1)). The following order for affinity to BZD receptors of different substituents in the R position was observed: phenyl > 6-methylpyridin-2-yl > 4-methylpyridin-2-yl > N-morpholinyl > cyclohexyl > pyridin-2-yl. This pattern suggests that the compounds with flat substituents in the R position exhibit higher affinity than the compounds with flexible groups. It seems that planarity can improve hydrophobic interaction in the binding site, which is consistent with Cook's model findings (Zhang et al., 1995[22]).

### Conformational analysis

Conformational analysis of the designed structures and diazepam, a reference compound, was performed by AM1 calculation using Hyperchem software. In Figure 1a(Fig. 1), the structures of the designed compound and diazepam are shown. Figure 1b(Fig. 1) demonstrates the superimposition of compound **6a**, the most potent synthesized analogue in radioligand binding assay, and diazepam. Obviously, the main BZD pharmacophores including the aromatic rings, proton accepting groups, and nitrogen atoms in position 3 of the 1,3,4-oxadiazole ring and carbonyl of the benzodiazepine were well matched.

Figure 1c(Fig. 1) represents locating of the compound **6a** in Cook's model (Zhang et al., 1995)[22]. Cook's model is a pharmacophore/ topological receptor model based on the binding affinity of rigid ligands to BZD/ GABA_A _receptor sites. The two aromatic rings in this compound and diazepam fitted properly in the L_2_ and L_3_ pockets and the proton accepting and donating groups had a suitable orientation for effective hydrogen bonding. In addition, a lipophilic ring linked to an oxadiazole residue with an amide bond could fit in L_1_ pocket. The other lipophilic pocket between the steric repulsive site (S_4_ and S_5_) enhances binding affinity to the BZD receptors. 

### Molecular modeling (docking) studies

**Figure 3:**
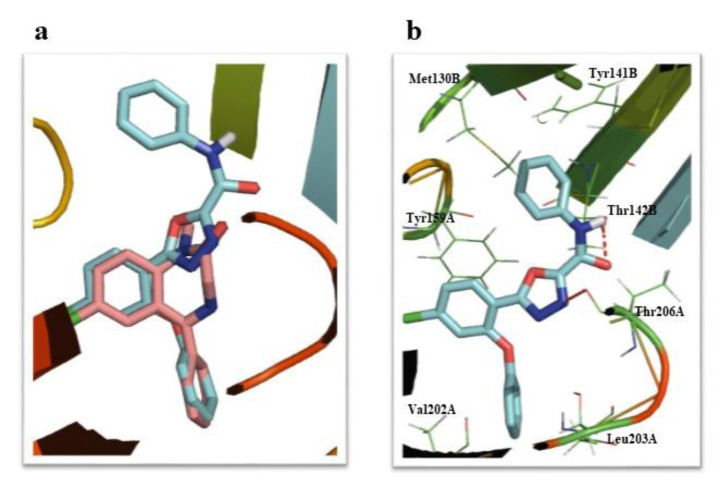
a: Superposed structures of diazepam (orange) and 6a (blue) inside the diazepam-binding pocket of GABA_A_ receptor (α1β2γ2). b: Interaction of 6a with amino acids of the diazepam-binding pocket of GABA_A_ receptor (α1β2γ2).

As shown in Table 1(Tab. 1), docking studies on the designed compounds confirmed that these compounds had a significant affinity to the diazepam-binding pocket of the GABA_A_ receptor (α1β2ϒ2). Compound **6a** was the most potent analogue in the radioligand binding assay and docking study was selected for the *in-vivo* tests. As displayed in Figure 3a(Fig. 3), it is clear that the main pharmacophores of **6a** with ΔG -11.39 (kcal/mol), and diazepam were in the same direction in the binding pocket of the GABA_A_ receptor. A π-π interaction could be formed between the phenyl ring of **6a** and α1 Tyr159. In addition, the phenoxy ring of this compound might bind with α1 Val202 and α1 Leu203 in a lipophilic pocket of the receptor. In addition, the amide group of **6a** has a suitable distance from the two amino acids of α1 Thr206 and β2 Thr142 for additional hydrogen bonds, and also the other phenyl ring of **6a** could interact with β2 Tyr141 and β2 Met130 in the active site (Figure 3b(Fig. 3)). Surprisingly, the results of docking and binding studies had a good correlation and the observed orders of substituents in the R position in both studies were similar.

### Pharmacological evaluations

#### PTZ-induced convulsion test and MES tests

Compound **6a** had the greatest affinity for the BZD receptors among the novel compounds. Therefore, pentylenetetrazol (PTZ) and maximal electroshock (MES) tests were used for the evaluation of the anticonvulsant activities of the selected compound. As shown in Table 2(Tab. 2), the anticonvulsant potency of the novel compound **6a** was lower than diazepam in both MES and PTZ tests (*P*<0.05). Flumazenil at doses of 10 mg/kg can antagonize the anticonvulsant activity of the compound in both tests. The antagonistic effect of flumazenil indicated that BZD receptors were highly involved in the anticonvulsant effects of compound **6a**.

#### Passive avoidance test

**Figure 4:**
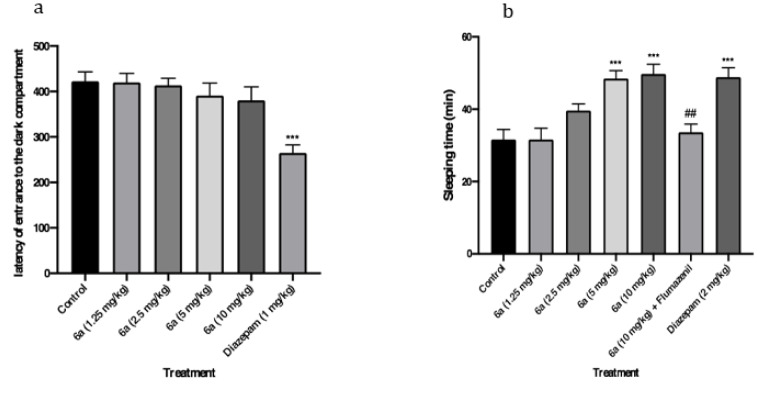
Effects of the novel compound (6a) on memory and sleeping time. (a) Passive avoidance test; the latency of entrance to the dark compartment in the testing day is shown. (b) Pentobarbital test; the sleeping time (duration of loss of righting reflex) is shown. Data are presented as mean ± SEM. In all groups n=8. *** represents *P*<0.001 compared to the control group. ## represents *P*<0.01 compared to the 6a (10 mg/kg) group.

Figure 4a(Fig. 4) presents the latency to enter the dark compartment in the passive avoidance test on testing day. Diazepam (1 mg/kg) was used as a positive control in the experiment and significantly decreased the latency compared to the negative control (vehicle; *P*<0.001). Therefore, diazepam showed anterograde amnesia in the passive avoidance test. However, compound **6a** at doses of 1.25, 2.5, 5, and 10 mg/kg did not show any significant effect in the passive avoidance test. It is obvious that compound **6a** had probably no effect on anterograde memory in the passive avoidance test.

#### Pentobarbital-induced sleep test

The duration of losing the righting reflex following injection of pentobarbital and compound **6a **is shown in Figure 4b(Fig. 4). Diazepam (2 mg/kg) and compound **6a** at the dose of 10 and 5 mg/kg increased the sleeping time (*P*<0.001, *P*<0.001). However, compound **6a **at doses of 1.25 and 2.5 mg/kg did not show any significant effect on the duration of sleeping time induced by pentobarbital. The hypnotic effect of compound **6a** was prevented by flumazenil (10 mg/kg) (*P*<0.001) revealing the role of BZD receptors in this effect.

#### Elevated-plus maze test

**Figure 5:**
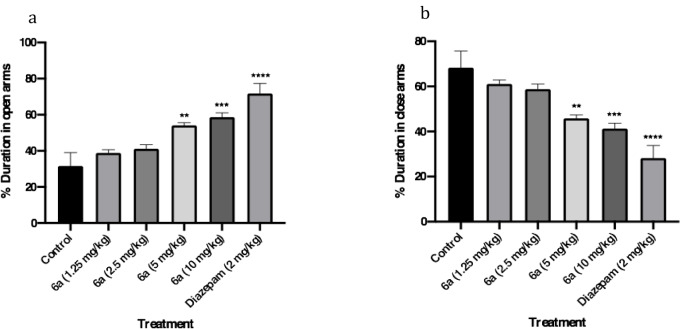
Anti-anxiety effects of the novel compound (6a) using EPM test. (a) percentage of duration in open arms, (b) percentage of duration in close arms. Data are presented as mean ± SEM. In all groups n=8. ** represents *P*<0.01 compared to the control group. *** represents *P*<0.001 compared to the control group. **** represents *P*<0.0001 compared to the control group.

The time spent on the open and close arms in the elevated plus-maze test, was measured over 10 minutes. Regarding Figure 5(Fig. 5), compound **6a** could increase the open arm time (OAT) and decrease the close arm time (CAT) at doses of 5 and 10 mg/kg. The potency of the anxiolytic action of compound **6a** was lower than diazepam (2 mg/kg).

See also the Supplementary data.

## Conclusion

In this study, we successfully designed and synthesized 2-substituted-5-(4-chloro-2-phenoxy) phenyl-1,3,4-oxadiazole derivatives as new benzodiazepine receptor agonists. By conformational analysis and superimposition of energy minima conformers of the designed novel compounds on diazepam, we found that the designed compounds could interfere properly with the GABA receptors. All of the synthesized compounds demonstrated an appropriate affinity for benzodiazepine receptors and most of them had a higher affinity than diazepam. Compound **6a** revealed potent hypnotic and weak anticonvulsant and anxiolytic activities with no impairment on learning and memory. The previous studies showed that sedation, anxiety, and the effect on memory are mediated through the GABA_A_ receptors with the subunits α_1, _α_2,3,_ and α_5, _respectively. It seems that the novel compound has a higher affinity to GABA_A_ receptors with subunit α_1_ than others. However_, _further studies such as the binding study of the designed compounds to the specific subunits of the GABA_A_ receptor are needed to prove this hypothesis. Subsequently, most of the currently used BZD agonists show unwanted effects on learning and memory, and we can use the scaffold of the novel compounds to design and develop other new agonists of benzodiazepine receptors.

## Experimental

### Materials and instrumentation 

Melting points (mp) were determined on the Electrothermal 9100 apparatus and uncorrected. Perkin Elmer 843 IR, and elemental analyzer (Costech, Italy) were used to obtain IR and elemental analysis, respectively. The ^1^HNMR spectra were acquired using a Bruker FT-500, 400 MHz instruments (Bruker Biosciences, USA), and chemical shifts (δ) were reported in ppm relative to tetramethylsilane, the internal standard. We used a Finnigan TSQ-70 mass spectrometer and HPLC Agilent system to get the mass spectra of the synthesized compounds. Electron-impact ionization was accomplished at ionizing energy of 70 eV. All chemicals for synthesis were supplied from Sigma-Aldrich and Merck Company (Germany) without further purification.

### 4-chloro-2-phenoxybenzoic acid (1) 

2,4-dichlorobenzoic acid (2 g, 10.52 mmol) and phenol (3.12 g, 33.19 mmol) were dissolved in DMF (20 mL). Potassium tert-butoxide (4.7 g, 41.88 mmol) and a catalytic amount of copper powder were added to the solution. The mixture was refluxed for 5h and became dark purple at the end. The reaction mixture was added to water (1.5 L) and while stirring, it was acidified with diluted hydrochloric acid (pH<7). The resulting precipitates were filtered and washed with water and recrystallized from ethanol 96 % to give 2.29 g (88 %) of **1**. mp:164-166 °C; IR:(KBr) ν (cm^-1^) 2887-3045 (OH), 1702 (C=O); MS m/z (%): 248.0 (M^+^, 30), 231.0 (5), 167.9 (20), 156.8 (80), 154.9 (100), 139.0 (30), 125.9 (15), 94.0 (30). 

### Ethyl 4-chloro-2-phenoxybenzoate (2) 

2 g (8.06 mmol) of **1** was dissolved in ethanol (15 mL) and concentrated sulfuric acid (0.5 mL) was added. The solution was refluxed for 24 h. Then, ethanol was evaporated and the remnant was alkalinized by NaOH 20 % after being cooled in an ice bath and then it was extracted with diethyl ether. The diethyl ether phase was washed first with aqueous NaOH 20 % and then with water. Then it was dried with anhydrous sodium sulfate and evaporated to give 1.77 g (80 %) of **2** as an oil; IR: (KBr) ν (cm^-1^) 1744 (C=O); MS m/z (%): 276.0 (M^+^, 70), 233 (60), 230.9 (85), 196.1 (35), 167.9 (70), 154.7 (100), 138.8 (80), 125.9 (30). 

### 4-chloro-2-phenoxybenzohydrazide (3) 

2 g (7.24 mmol) of **2** and 10 mL hydrazine hydrate (200 mmol) were added to 10 mL ethanol. The mixture was stirred for 12 h at room temperature. Afterward, the solvent was evaporated. The white precipitate was washed with diethyl ether and recrystallized from a mixture of ethanol and a few drops of water to give 8 g (84 %) of 3. mp: 100.5-102 °C; IR: (KBr) ν (cm^-1^) 3397, 3307 (NH_2_), 1647 (C=O); MS m/z (%): 262.0 (M^+^, 5), 233.1 (30), 231.0 (100), 195.9 (15), 168.0 (20), 139.0 (30), 76.9 (25). 

### Ethyl 2-(2-(4-chloro-2-phenoxybenzoyl)hydrazinyl)-2-oxoacetate (4) 

2 g (7.63 mmol) of **3** and triethylamine (2ml) were dissolved in THF (25 mL). Ethylchloroglyoxylate (1.8 ml) was slowly added via syringe at 0 °C. The mixture was stirred at room temperature for 3 h. The resulting precipitate was filtered and washed with water and recrystallized from ethanol 96 % to give 2.4 g (85 %) of **4**. mp: 185-187 °C; IR: (KBr) ν (cm^-1^) 3341, 3162 (NH), 1631, 1706, 1742 (C=O); LC-MS m/z: 385 (M+23).

### Ethyl 5-(4-chloro-2-phenoxyphenyl)-1,3,4 oxadiazole-2- carboxylate (5) 

2 g (5.52 mmol) of **4 **and P_2_O_5 _(3 g) were added to toluene (30 mL) and refluxed for 3 h. The toluene phase was evaporated and recrystallized from n-hexane to give 1.1 g (57 %) of 5. mp: 93.8-94.3 °C; IR: (KBr) ν (cm^-1^) 1744 (C=O); 500 MHz ^1^H-NMR (CDCl_3_): δ (ppm) 1.5 (t, 3H, CH_3_, *J* = 7.18 Hz), 4.6 (q, 2H, CH_2_), 7.02 (d, 1H, phenyl-H_3_, *J* = 1.9 Hz), 7.14 (d, 2H, phenoxy-H_2',6'_, *J* = 8.2 Hz), 7.25-7.28 (m, 2H, phenyl-H_5_, phenoxy-H_4'_), 7.45 (t, 2H, phenoxy-H_3',5'_, *J *= 9.8 Hz), 8.1 (d, 1H, phenyl-H_6_, *J* = 8.5 Hz), 100 MHz C-NMR (CDCl_3_): δ (ppm)14.08, 63.39, 113.93, 118.10, 121.01, 123.70, 125.59, 126.18, 128.12, 131.05, 131.19, 134.05, 151.48, 154.41, 155.31, 156.74, 164.69; GC-S m/z (%): 344.1 (M^+^, 70), 343.1 (75), 315.1 (55), 299.1 (15), 271.1 (15), 91.1 (80), 77.1 (100). Anal. Calcd for C_17_H_13_ClN_2_O_4_: C, 59.23, H, 3.80, N, 8.13, Found: C, 59.06, H, 3.75, N, 8.21.

### General procedure for the synthesis of the compounds 6a-f

7.02 mmol proper amine was added to a mixture of **5** (0.5 g, 1.45 mmol) and 0.76 g AlCl_3_ (5.7 mmol) in dichloromethane (15 mL). We stirred the mixture for 3 h at room temperature. Then, we added water and extracted the final mixture with dichloromethane. The dichloromethane phase was dried with anhydrous sodium sulfate, evaporated, and then recrystallized from ethanol 96 % to give corresponding amides **6a-f.**

#### 5-(4-chloro-2-phenoxyphenyl)-N-phenyl-1,3,4-oxadiazole-2-carboxamide (6a) 

Yield: 95 %, mp: 183.2-184.3 °C; IR: (KBr) ν (cm^-1^) 3132 (N-H), 1698 (C=O); 500 MHz ^1^H-NMR (CDCl_3_): δ ppm 6.975 (d, 1H, phenyl-H_3_, *J* = 2.5 Hz), 7.12 (dd, 2H, phenoxy-H_2', 6'_, *J* = 1.5, 11 Hz), 7.2-7.3 (m, 3H, phenyl-H_5_, phenoxy-H_4'_, anilin-H_4”_), 7.38-7.44 (m, 4H, phenoxy-H_3', 5'_, anilin-H_3”, 5”_), 7.67 (dd, 2H, anilin-H_2”, 6”_, *J* = 1.5, 11 Hz), 8.08 (d, 1H, phenyl-H_6_, *J* = 10.5 Hz), 8.6 (1H, NH); 100 MHz C-NMR (CDCl_3_): δ ppm 110.55, 111.57, 117.72, 119.02, 119.58, 122.60, 124.09, 129.23, 130.76, 138.55, 138.86, 147.94, 149.99, 154.04, 155.55, 156.71, 157.17, 164.57; LC-MS m/z: 414 (M+23). Anal. Calcd for C_21_H_14_ClN_3_O_3_: C, 64.38, H, 3.60, N, 10.72. Found: C, 64.56, H, 3.61, N, 10.54.

#### 5-(4-chloro-2-phenoxyphenyl)-N-(pyridin-2-yl)-1,3,4-oxadiazole-2-carboxamide (6b) 

Yield: 96 %, mp: 198-200 °C; IR: (KBr) ν (cm^-1^) 3372 (N-H), 1711 (C=O); 500 MHz ^1^H-NMR (CDCl_3_): δ ppm: 6.97 (d, 1H, phenyl-H_3_, *J* = 2.5 Hz), 7.11-7.16 (m, 3H, phenoxy-H_2',6'_, pyridin_-H5”_), 7.2-7.26 (m, 2H, pheyl-H_5_, phenoxy-H_4'_), 7.34 (t, 2H, phenoxy-H_3',5'_, *J* = 8.8 Hz), 7.8 (t, 1H, pyridin-H_4”_, *J* = 9 Hz), 8.09 (d, 1H, phenyl-H_6_, *J* = 10 Hz), 8.26-8.28 (m, 1H, pyridin-H_6”_), 8.37-8.39 (m, 1H, Pyridin-H_3”_), 9.6 (1H, NH). 100 MHz C-NMR (CDCl_3_): δ ppm 110.45, 111.57, 117.70, 119.05, 119.58, 122.60, 124.09, 129.25, 130.76, 138.54, 138.46, 147.94, 149.49, 154.04, 155.94, 156.21, 157.04, 162.52; LC-MS m/z: 415 (M+23). Anal. Calcd for C_20_H_13_ClN_4_O_3_: C, 61.16, H, 3.34, N, 14.26. Found: C, 61.34, H, 3.27, N, 14.19.

#### 5-(4-chloro-2-phenoxyphenyl)-N-(6-methylpyridin-2-yl)-1,3,4-oxadiazole-2-carboxamide (6c) 

Yield: 93 %, mp: 183.2-184.3 °C; IR: (KBr) ν (cm^-1^) 3376 (N-H), 1716 (C=O); 500 MHz ^1^H-NMR (CDCl_3_): δ ppm: 2.5 (s, 3H, CH_3_), 6.96 (d, 1H, Phenyl-H_3_, *J* = 2.5 Hz), 6.99 (d, 1H, methyl pyridin-H_4”_, *J* = 9 Hz), 7.12 (d, 2H, phenoxy-H_2', 6'_, *J* = 10 Hz), 7.2-7.3(m, 2H, phenyl-H_5_, phenoxy-H_4'_), 7.41 (t, 2H, phenoxy-H_3', 5'_, *J* = 9.5 Hz), 7.65 (t, 1H, methyl pyridin-H_5”_, *J* = 10 Hz), 8.06 (d, 1H, methyl pyridin-H_6”_, *J* = 10 Hz), 8.09 (d, 1H, phenyl-H_6_, *J* = 11 Hz), 9.4 (1H, NH); 100 MHz C-NMR (CDCl_3_): δ ppm 23.89, 110.45, 111.57, 117.70, 119.05, 119.58, 122.60, 124.09, 129.23, 130.79, 138.55, 138.86, 147.94, 149.69, 154.04, 155.95, 156.41, 157.14, 164.57; LC-MS m/z: 429 (M+23). Anal. Calcd for C_21_H_15_ClN_4_O_3_: C, 62.00, H, 3.72, N, 13.77. Found: C, 62.11, H, 3.76, N, 13.66.

#### 5-(4-chloro-2-phenoxyphenyl)-N-(4-methylpyridin-2-yl)-1,3,4-oxadiazole-2-carboxamide (6d) 

Yield: 90 %, mp: 188.2-189.1 °C; IR: (KBr) ν (cm^-1^) 3367 (N-H), 1709 (C=O); 500 MHz ^1^H-NMR (CDCl_3_): δ ppm: 2.4 (s, 3H, CH_3_), 6.8-7 (m, 2H, phenyl-H_3_, methyl pyridin-H_4”_), 7.12 (d, 2H, phenoxy-H_2',6'_, *J* = 7.6 Hz), 7.2-7.29 (m, 2H, Phenyl-H_5_, Phenoxy-H_4'_), 7.42 (t, 2H, Phenoxy-H_3',5'_, *J* = 9 Hz), 8.08 (d, 1H, Phenyl-H_6_, *J* = 10.5 Hz), 8.12 (bs, 1H, methyl pyridin-H_6”_), 8.22 (d, 1H, methyl pyridin-H_3”_, *J* = 6 Hz), 9.4 (1H, NH); 100 MHz C-NMR (CDCl_3_): δ ppm 22.59, 110.55, 111.37, 117.70, 119.02, 119.58, 122.60, 124.09, 129.23, 130.76, 138.55, 138.86, 147.94, 149.99, 154.04, 155.95, 156.11, 157.17, 163.57; LC-MS m/z: 429 (M+23). Anal. Calcd for C_21_H_15_ClN_4_O_3_: C, 62.00, H, 3.72, N, 13.77. Found: C, 61.94, H, 3.75, N, 13.81.

#### 5-(4-chloro-2-phenoxyphenyl)-N-morpholino-1,3,4-oxadiazole-2-carboxamide (6e) 

Yield: 88 %, mp: 159-161 °C; IR: (KBr) ν (cm^-1^) 3186 (N-H), 1625 (C=O); 500 MHz ^1^H-NMR (CDCl_3_): δ ppm: 2.86 (t, 4H, CH_2_-H_2”_, 6”, *J* = 5.5 Hz), 3.82 (t, 4H, CH_2_-H_3”, 5”_, *J* = 7 Hz), 6.8 (d, 1H, phenyl-H_3_, *J* = 2 Hz), 7.15 (d, 2H, phenoxy-H_2', 6'_, *J* = 6.5 Hz), 7.19 (dd, 1H, phenyl-H_5_, *J* = 2, 8.5 Hz ), 7.29 (t, 1H, phenoxy-H_4'_, *J* = 9.5 Hz), 7.48 (t, 2H, phenoxy-H_3',5'_, *J* = 10.25 Hz), 8.06 (1H, NH), 8.19 (d, 1H, Phenyl-H_6_, *J* = 10.5 Hz); 100 MHz C-NMR (CDCl_3_): δ ppm 55.69, 66.22, 112.48, 118.99, 119.85, 123.70, 125.04, 130.25, 131.68, 139.86, 151.00, 155.18, 156.96, 157.66, 164.18; LC-MS m/z : 423 (M+23). Anal. Calcd for C_19_H_17_ClN_4_O_4_: C, 56.94, H, 4.28, N, 13.98. Found: C, 57.10, H, 4.22, N, 13.91.

#### 5-(4-chloro-2-phenoxyphenyl)-N-cyclohexyl-1,3,4-oxadiazole-2-carboxamide (6f) 

Yield: 94 %, mp: 132-133 °C; IR: (KBr) ν (cm^-1^) 3305 (N-H), 1672 (C=O); 500 MHz ^1^H-NMR (CDCl_3_): δ ppm: 1.23-1.42 (m, 5H, cyclohexylamine), 1.64-1.66 (m, 1H, cyclohexylamine), 1.74-1.78 (m, 2H, cyclohexylamin-H_2”,6”_), 1.99-2.02 (m, 2H, cyclohexylamin-H_2”,6”_), 3.92-3.98 (m, 1H, cyclohexylamin-H_1”_), 6.95 (d, 2H, Phenyl-H_3_, NH, *J* = 1.9 Hz ), 7.08 (d, 2H, Phenoxy-H_2',6'_, *J* = 8.5 Hz), 7.19-7.22 (m, 2H, phenyl-H_5_, phenoxy-H_4'_), 7.38 (t, 2H, phenoxy-H_3',5'_, *J* = 11 Hz), 8.02 (d, 1H, phenyl-H_6_, *J* = 8.5 Hz); 100 MHz C-NMR (CDCl_3_): δ ppm 24.28, 25.02, 34.08, 53.48, 112.63, 119.03, 119.78, 123.75, 124.99, 130.22, 130.57, 131.86, 139.90, 154.35, 155.24, 156.68, 156.87, 164.22; LC-MS m/z : 420 (M+23). Anal. Calcd for C_21_H_20_ClN_3_O_3_: C, 63.40, H, 5.07, N, 10.56. Found: C, 63.50, H, 4.96, N, 10.45.

### Radioligand receptor binding assay

Competitive binding studies were used to determine the concentration of the ligands required to reduce the specific binding of [^3^H]-flumazenil by 50 % (IC_50_) according to our previous study. All of the binding assays were performed in triplicates in a total volume of 500 µL at 30 °C for 35 min (Ahmadi et al., 2013[1]).

### Conformational analysis

HyperChem8 software (Hypercube, Inc.) was applied for conformational analysis of the synthesized compounds and diazepam. We used MMX force field method followed by AM_1_ calculations to find the optimum conformations of the novel compounds and diazepam. Then, we superimposed the novel compounds with diazepam.

### Molecular modeling (docking) studies

The homology model of the diazepam-bound GABA_A_ receptor (α1β2ϒ2) was retrieved from the supplementary material of the previously reported paper developed by Richter et al. (2012[15]). The Lamarckian genetic algorithm search method implemented in AutoDock 4.2.3 software (http://autodock.scripps.edu) was applied to investigate the structures of the compounds. The receptor was allowed to be rigid while the ligands were allowed to be flexible. Then, polar Hydrogens with Kollman united atom partial charges were added to the individual protein atoms. Each structure is converted to the PDBQT format file using AutoDockTools version1.5.6rc3 (http://mgltools.scripps.edu). Finally, the energy was minimized under the MM+ method with HyperChem 8 software. A docking grid box with 40, 40, and 40 points was constructed in 42.820, 44.4360, and 6.8690 directions, and the number of generations and the maximum number of energy evaluations were set to 100 and 2,700,000, respectively. We used Pymol software (version 1.5.0.1) with a root mean square deviation (RMSD) of 0.5 Å to cluster the docking results (http://pymol.findmysoft.com) (Faizi et al., 2015[6]).

### Pharmacological evaluations

#### Animals and drugs

For pharmacological evaluations of the selected novel compound (**6a**), we used male NMRI albino mice (Pasteur Institute, Iran) weighing 18-25 g (n = 8 in each group). The mice were kept in a facility with a 12-hour light/dark cycle and a controlled temperature (22 ± 2 °C) and humidity (50 ± 5 %) and they had free access to a standard mouse diet and tap water in the facility. For the mice to get adapted to the lab environment, we transferred them to the lab 60 minutes before the tests started. Mice were randomly selected for all the experimental groups and each subject was tested only once for the pharmacological evaluations. At the beginning of the experiments, the proposal of the study was approved by the Ethical Committee of Shahid Beheshti University of Medical Sciences (Ethics code IR.SBMU.PHARMACY.REC.1399.300). 

We performed all the experiments based on the protocols of the National Institutes of Health (NIH) Principles of Laboratory Animal Care. The novel compound **6a**, diazepam as recommended BZD agonist, and flumazenil as a regular antagonist of BZD receptors were dissolved in a mixture of DMSO and water (1:10) and administered 5 ml/kg; i.p.; 30 minutes before the tests. Distilled water was used as a solvent (vehicle) of PTZ and pentobarbital. Both, PTZ and pentobarbital were injected i.p. 5 ml/kg. 

#### PTZ-induced convulsion and MES tests

To assess the anticonvulsant activity of the novel compound **6a,** we used PTZ-induced convulsion and MES tests (Guerrini et al., 2008[10]; Lankau et al., 2007[13]). In both models, the ability of the novel compound to protect mice against induced seizures was evaluated. Animals were closely observed for 30 minutes after injection of the lethal dose of PTZ (100 mg/kg) and the number of dead mice after convulsion was reported. In the MES test, each subject was observed closely and the incidence of hind limb tonic extension (HLTE) in the subjects after application of MES (60 Hz, 37.2 mA, and 0.25 s) was reported. For the incidence of HLTE, the electrical current was applied through ear electrodes and it was observed for 30 seconds.

#### Passive avoidance test

Anterograde amnesia induced by the selected novel compound **6a** and diazepam (1 mg/kg, *i.p.*), a BZD with amnestic effects, was evaluated by a step-throw passive avoidance test (Khoramjouy et al., 2021[11]). The passive avoidance cage (Malek Teb Co., Tehran) consists of two different compartments separated by a guillotine door. One of the compartments was black and dark while the other one was white and lighted. For the training trial, each mouse was placed in the white and light compartment and facing away from the guillotine door for 30 seconds. Next, the guillotine door was opened and the mouse was able to freely enter the black and dark compartment. When the mouse entered the black and dark compartment with four paws, it received an electric unpleasant stimulus (0.5 mA, 2 seconds) through the grid floor. On the testing day (after 24 hours), we placed the mouse in the lighted compartment and there was no electrical stimulus. We reported the latency to enter the black and dark compartment while the guillotine door was open.

#### Pentobarbital-induced sleep test

The duration of the loss of the righting reflex was considered as the sleeping time. After 30 min of administration, the novel compound **6a** and diazepam (2 mg/kg), and pentobarbital (40 mg/kg i.p.) were injected to animals. For prevention of hypothermia during the experiment, animals were placed on an electric blanket (Khoramjouy et al., 2021[11]).

#### Elevated plus-maze test

The elevated plus-maze device was made up of Plexiglas, and it contained two open arms (30×5×0.5 cm) and two closed arms (30×5×35 cm) with an open roof. They were arranged so that the open arms were opposite to close arms. The entire maze was elevated at a height of 1 meter. The mice were individually placed in the central square of the maze (5×5 cm) where all four arms were connected together after 30 minutes of intraperitoneal (*i.p*) administration of the test compounds. The time spent in the open and closed arms was recorded. Finally, the effects of the compounds **6a **and diazepam (2 mg/kg, *i.p.*), the reference drug, were evaluated (Khoramjouy et al., 2021[11]).

### Data analysis

K_d_ and B_max_ in radioligand binding assay were calculated by non-linear regression analysis of the saturation and competitive curve data (Program Prism, Graph Pad, San Diego, CA). The IC_50_s of the novel compounds in binding studies were determined using one-way ANOVA with Tukey's HSD post-hoc test. In PTZ and MES tests, the probit-regression method and SPSS software (Chicago, IL; version 13) were used to determine ED_50 _of the selected novel compound **6a** and diazepam. The difference between the ED_50_ of the novel compound and diazepam was statistically analyzed using Fisher's exact probability test. In PTZ and MES tests, the data were presented as a mean with 95% confidence intervals. In the rest of the pharmacological evaluations, one-way ANOVA with Tukey's HSD post-hoc test was used. All the data were presented as mean ± SEM and *P*<0.05 was considered statistically significant.

## Notes

Sayyed Abbas Tabatabai and Mehrdad Faizi (Department of Pharmacology and Toxicology, School of Pharmacy, Shahid Beheshti University of Medical Sciences, Tehran, Iran; E-mail: m.faizi@sbmu.ac.ir) contributed equally as corresponding author.

## Acknowledgement

Part of this work was supported by a grant from Shahid Beheshti University of Medical Sciences [Grant No. 26170].

## Supplementary Material

Supplementary data

## Figures and Tables

**Table 1:**
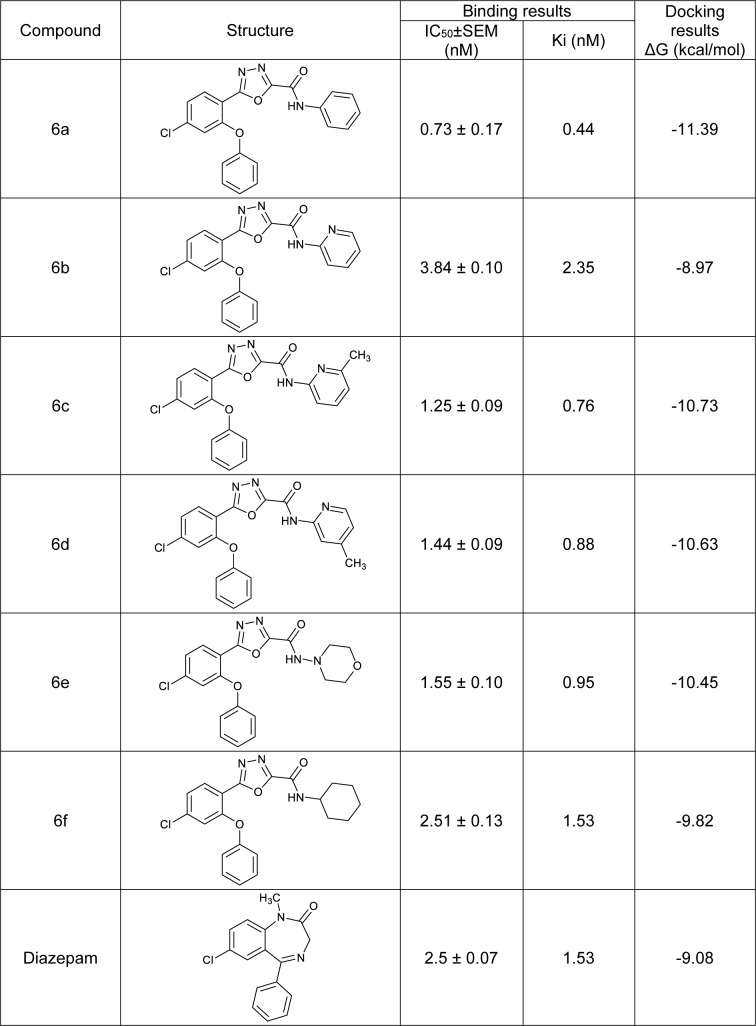
*In vitro* binding affinities (IC_50_, ki) and docking study results (ΔG) of the novel compounds

**Table 2:**
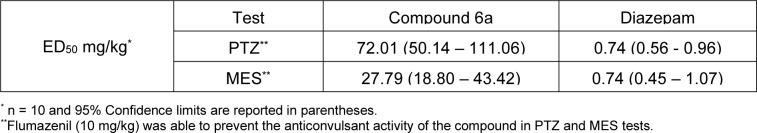
PTZ-induced lethal convulsion and MES tests of the compound 6a
