# Supplementary material for: Novel 2-substituted-5-(4-chloro-2-phenoxy)phenyl-1,3,4-oxadiazole derivatives, ligands of GABAA/benzodiazepine receptor complex: Design, synthesis, radioligand binding assay, and pharmacological evaluation
Source: EXCLI J. 2023 Feb 20;22:250–62. doi: 10.17179/excli2022-5639 (PMC10043390; doi:10.17179/excli2022-5639)
Supplement: Supplementary data [file EXCLI-22-250-s-001.pdf]

EXCLI Journal 2023;22:250-262 – ISSN 1611-2156
Received: November 28, 2022, accepted: February 02, 2023, published: February 20, 2023

Supplementary data to:

Original article:

NOVEL 2-SUBSTITUTED-5-(4-CHLORO-2-PHENOXY)PHENYL-1,3,4-OXADIAZOLE DERIVATIVES, LIGANDS OF GABA$_A$/BENZODIAZEPINE RECEPTOR COMPLEX: DESIGN, SYNTHESIS, RADIOLIGAND BINDING ASSAY, AND PHARMACOLOGICAL EVALUATION

Elham Rezaee[1], Fatemeh Ahmadi[1], Mahsa Shabaninia[2], Mona Khoramjouy[3], Zahra Azizi Farsani[4], Soraya Shahhosseini[1], Sayyed Abbas Tabatabai[1*], Mehrdad Faizi[4**]

[1] Department of Pharmaceutical Chemistry, School of Pharmacy, Shahid Beheshti University of Medical Sciences, Tehran, Iran
[2] Premier Care Long Term Care Pharmacy, North Little Rock, Arkansas, USA
[3] Phytochemistry Research Center, Shahid Beheshti University of Medical Sciences, Tehran, Iran
[4] Department of Pharmacology and Toxicology, School of Pharmacy, Shahid Beheshti University of Medical Sciences, Tehran, Iran

* **Corresponding author:** Sayyed Abbas Tabatabai, Department of Pharmaceutical Chemistry, School of Pharmacy, Shahid Beheshti University of Medical Sciences, Tehran, Iran. E-mail: sa_tabatabai@sbmu.ac.ir

** **Co-corresponding author:** Mehrdad Faizi, Department of Pharmacology and Toxicology, School of Pharmacy, Shahid Beheshti University of Medical Sciences, Tehran, Iran. E-mail: m.faizi@sbmu.ac.ir

https://dx.doi.org/10.17179/excli2022-5639

This is an Open Access article distributed under the terms of the Creative Commons Attribution License (http://creativecommons.org/licenses/by/4.0/).

EXCLI Journal 2023;22:250-262 – ISSN 1611-2156
Received: November 28, 2022, accepted: February 02, 2023, published: February 20, 2023

Supplementary data (1) to Table 1: Determining the time to reach the steady state in radioligand receptor binding assay. All data are reported in triplicate (n=3) and based on the mean ± standard deviation from the mean (Mean ± SEM)

Incubation time (min)	TB (cpm)	NSB (cpm)	SB (cpm)
0	0	0	0
10	456.6±60.2	90.3±2.8	367±59.3
20	516±9.8	104±1	412±10.58
25	537±32.5	104.3±2.08	433.6±20.6
30	577.6±79.8	102.3±9.86	455.3±70.4
40	577.6±47.3	99.6±9.29	458±50.06

TB = Total binding
cpm = Count per minute
NSB = Non-specific binding
SB = Specific binding

Supplementary data (2) to Table 1: Percent of total binding based on increasing amount of tissue in radioligand receptor binding assay. All data are presented as triplicates and based on the mean ± standard deviation of the mean. (Mean ± SEM)

Protein concentration (μg)	TB (cpm)	TA (cpm)	(TB×100)/TA
0	0	0	0
50	194.3±12.5	3974	4.85±0.27
100	316±61.7	3974	7.9±1.56
150	457.6±46.7	3974	11.48±1.14
200	517.6±14.04	3974	12.98±0.35
250	551.3±105.02	3974	13.83±2.6
300	580±12.28	3974	14.55±0.3

TB = Total binding
cpm = Count per minute
TA = Total added

EXCLI Journal 2023;22:250-262 – ISSN 1611-2156
Received: November 28, 2022, accepted: February 02, 2023, published: February 20, 2023

Supplementary data (3) to Table 1: Determination of specific binding against different concentrations of labeled flumazenil in radioligand receptor binding assay. All data are reported in triplicate (n=3) and based on the mean ± standard deviation from the mean (Mean ± SEM)

^{3}H-flumazenil (nM)	TB (cpm)	NSB (cpm)	SB (cpm)
0.97	2129.67±11.2	153.5.3±5.3	1976.7±7.7
0.86	2056.6±37.1	149.67±3.53	1907±33.65
0.63	1836±107.3	141.33±3.48	1695±110.6
0.4	1358.33±6.39	118.33±9.83	1240.3±3.93
0.28	776.3±61.9	113.3±8.46	663±69.6
0.17	429±67.1	101.67±5.5	327.36±4.9
0.05	240±16.09	86.67±5.79	153.33±21.4

TB = Total binding
cpm = Count per minute
NSB = Non-specific binding
SB = Specific binding

Supplementary data (4a) to Table 1: The effect of Diazepam concentration on the binding interval of triturated [3H]flumazenil

log[L]	Specific binding (cpm)
-11.0	0.5833±0.0088
-10.0	0.5467±0.0234
-9.5	0.4967±0.0033
-9.0	0.4467±0.0033
-8.5	0.3533±0.0033
-8.0	0.2633±0.0133
-7.0	0.1733±0.0120

cpm = Count per minute

EXCLI Journal 2023;22:250-262 – ISSN 1611-2156
Received: November 28, 2022, accepted: February 02, 2023, published: February 20, 2023

Supplementary data (4b) to Table 1: Effect of concentration of 5-(4-chloro-2-phenoxyphenyl)-N-cyclohexyl-4,3,1-oxadiazole-2-carboxamide (6f) on the binding interval of tritiated [3H]flumazenil. All data are reported in triplicate (n=3) and based on the mean ± standard deviation from the mean

log[L]	Specific binding (cpm)
-11.0	0.5933±0.0207
-10.0	0.6133±0.0296
-9.5	0.5500±0.0156
-9.0	0.4533±0.0148
-8.5	0.4000±0.0118
-8.0	0.3533±0.0189
-7.0	0.2133±0.0272

cpm = Count per minute

Supplementary data (5) to Table 1: Concentration of 5-(4-chloro-2-phenoxyphenyl)-N-(pyridin-2-yl)-1,3,4-oxadiazole-2-carboxamide (6b) on the binding interval of tritium-labeled [3H]flumazenil. All data are reported in triplicate (n=3) and based on the mean ± standard deviation from the mean

log[L]	Specific binding (cpm)
-11.0	0.6433±0.0122
-10.0	0.5900±0.0118
-9.5	0.5467±0.0068
-9.0	0.5233±0.0180
-8.5	0.4267±0.0090
-8.0	0.3733±0.0223
-7.0	0.2333±0.0148

cpm = Count per minute

EXCLI Journal 2023;22:250-262 – ISSN 1611-2156
Received: November 28, 2022, accepted: February 02, 2023, published: February 20, 2023

Supplementary data (6) to Table 1: Effect of concentration of 5-(4-chloro-2-phenoxyphenyl)-N-(6-methylpyridin-2-yl)-1,3,4-oxadiazole-2-carboxamide (6c) on the binding interval of tritium[3H]flumazenil. All data are reported in triplicate (n=3) and based on the mean ± standard deviation from the mean

log[L]	Specific binding (cpm)
-11.0	0.5733±0.0207
-10.0	0.5700±0.0156
-9.5	0.5367±0.0122
-9.0	0.4467±0.0207
-8.5	0.3767±0.0090
-8.0	0.3100±0.0102
-7.0	0.3000±0.0059

cpm = Count per minute

Supplementary data (7) to Table 1: Effect of concentration of 5-(4-chloro-2-phenoxyphenyl)-N-(4-methylpyridin-2-yl)-1,3,4-oxadiazole-2-carboxamide (6d) on the binding interval of tritium[3H]flumazenil . All data are reported in triplicate (n=3) and based on the mean ± standard deviation from the mean

log[L]	Specific binding (cpm)
-11.0	0.5733±0.0207
-10.0	0.5700±0.0156
-9.5	0.5367±0.0122
-9.0	0.4467±0.0207
-8.5	0.3767±0.0090
-8.0	0.3100±0.0102
-7.0	0.3000±0.0059

cpm = Count per minute

Supplementary data (8) to Table 1: Effect of concentration of 5-(4-chloro-2-phenoxyphenyl)-N-(4-morpholino)-1,3,4-oxadiazole-2-carboxamide (6e) on the binding interval of tritiated [3H]flumazenil. All data are reported in triplicate (n=3) and based on the mean ± standard deviation from the mean

log[L]	Specific binding (cpm)
-11.0	0.6967±0.0148
-10.0	0.6667±0.0148
-9.5	0.6033±0.0189
-9.0	0.5867±0.0090
-8.5	0.3600±0.0060
-8.0	0.3067±0.0090
-7.0	0.2900±0.0059

cpm = Count per minute

EXCLI Journal 2023;22:250-262 – ISSN 1611-2156
Received: November 28, 2022, accepted: February 02, 2023, published: February 20, 2023

Supplementary data (9) to Table 1: Effect of concentration of 5-(4-chloro-2-phenoxyphenyl)-N-phenyl-1,3,4-oxadiazole-2-carboxamide (6a) on the binding interval of tritium-labeled flumazenil. [3H]Flumazenil, all data in triplicate (n= 3) and are reported based on the mean ± standard deviation from the mean

log[L]	Specific binding (cpm)
-11.0	0.6267±0.0034
-10.0	0.5800±0.0156
-9.5	0.5100±0.0156
-9.0	0.4667±0.0090
-8.5	0.4200±0.0102
-8.0	0.4000±0.0412
-7.0	0.3167±0.0378

cpm = Count per minute

EXCLI Journal 2023;22:250-262 – ISSN 1611-2156
Received: November 28, 2022, accepted: February 02, 2023, published: February 20, 2023

Supplementary data (10): Analysis data (IR, Mass) of 4-chloro-2-phenoxybenzoic acid (1)

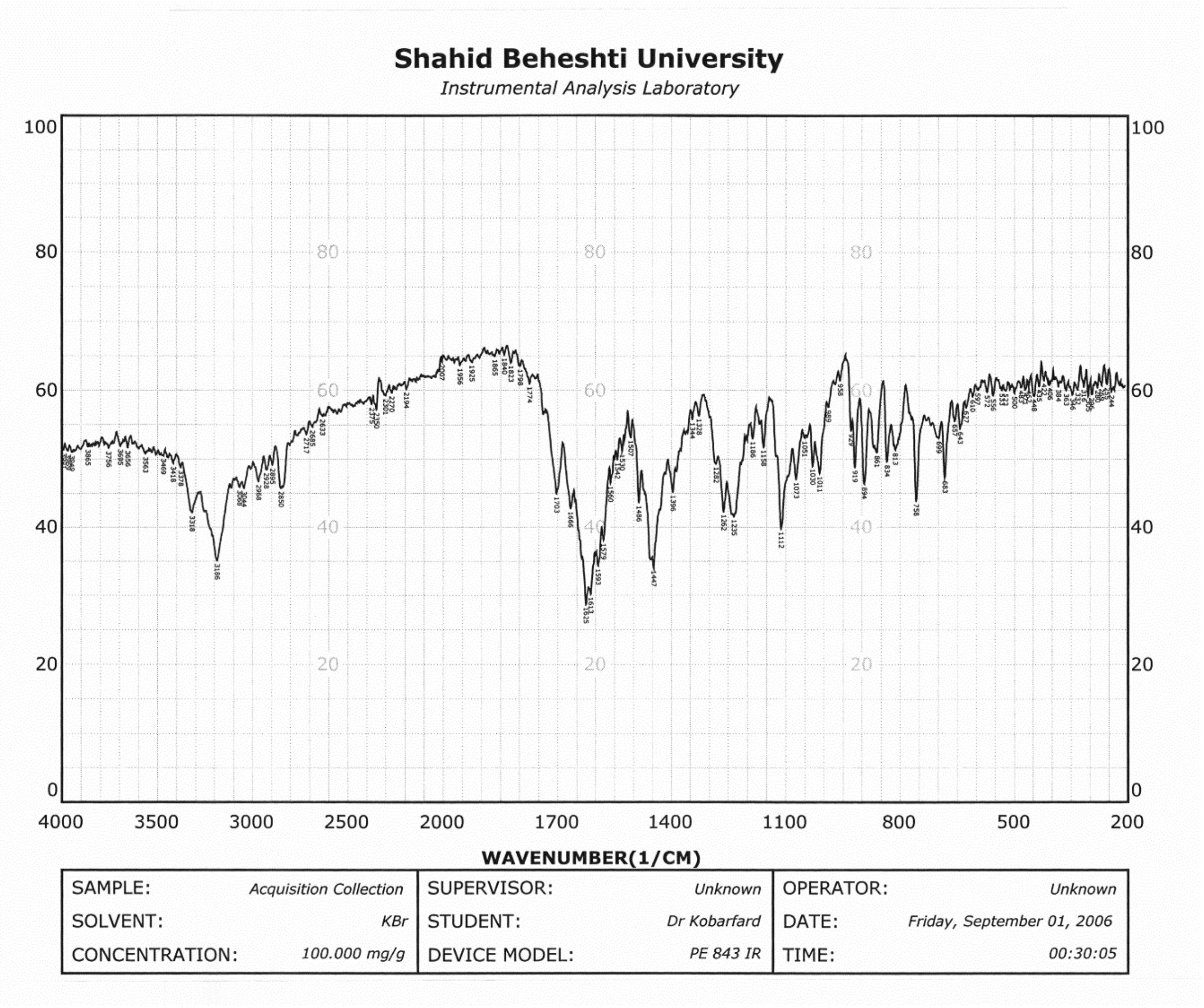

EXCLI Journal 2023;22:250-262 – ISSN 1611-2156
Received: November 28, 2022, accepted: February 02, 2023, published: February 20, 2023

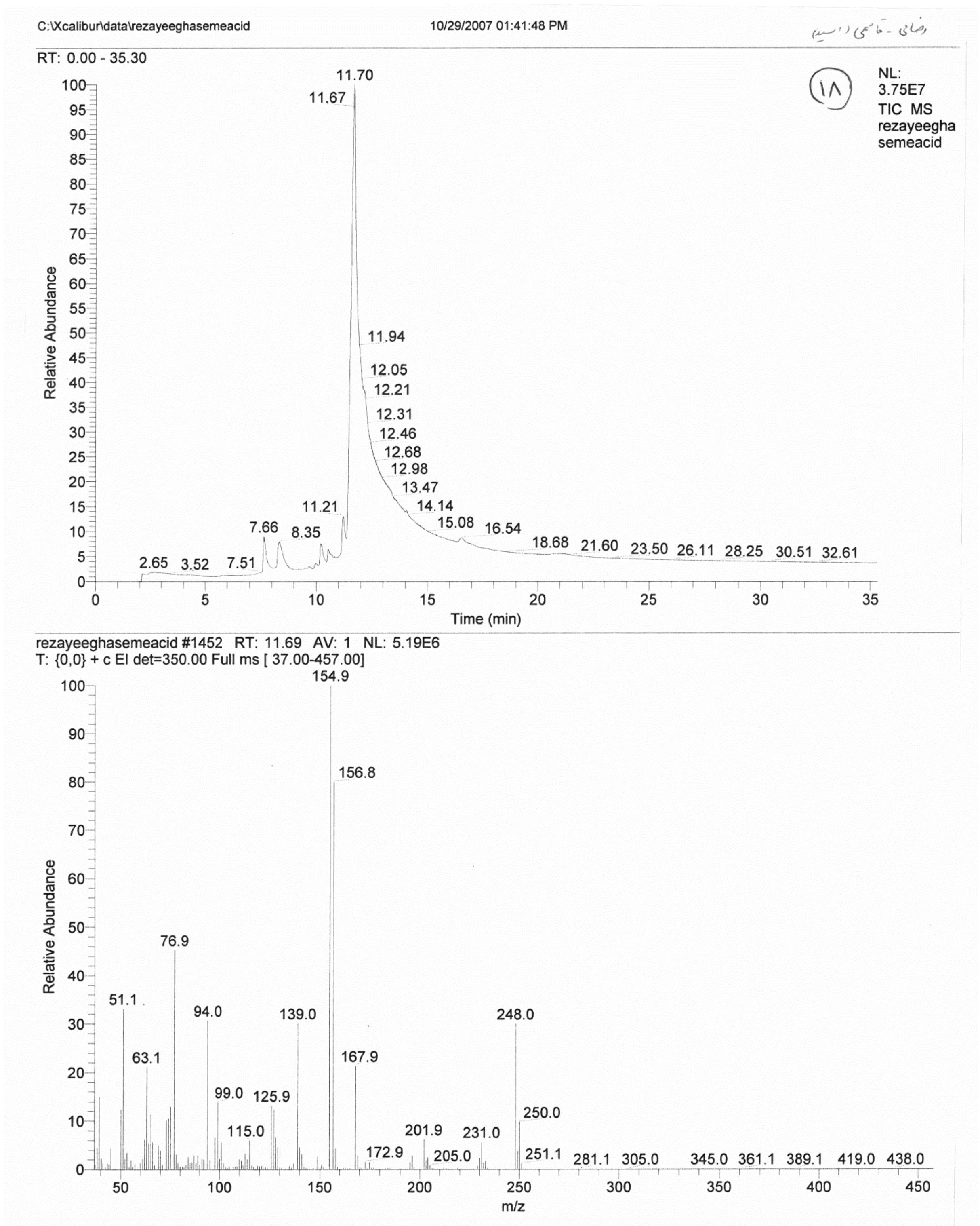

EXCLI Journal 2023;22:250-262 – ISSN 1611-2156
Received: November 28, 2022, accepted: February 02, 2023, published: February 20, 2023

Supplementary data (11): Analysis data (IR, Mass) of Ethyl 4-chloro-2-phenoxybenzoate (2)

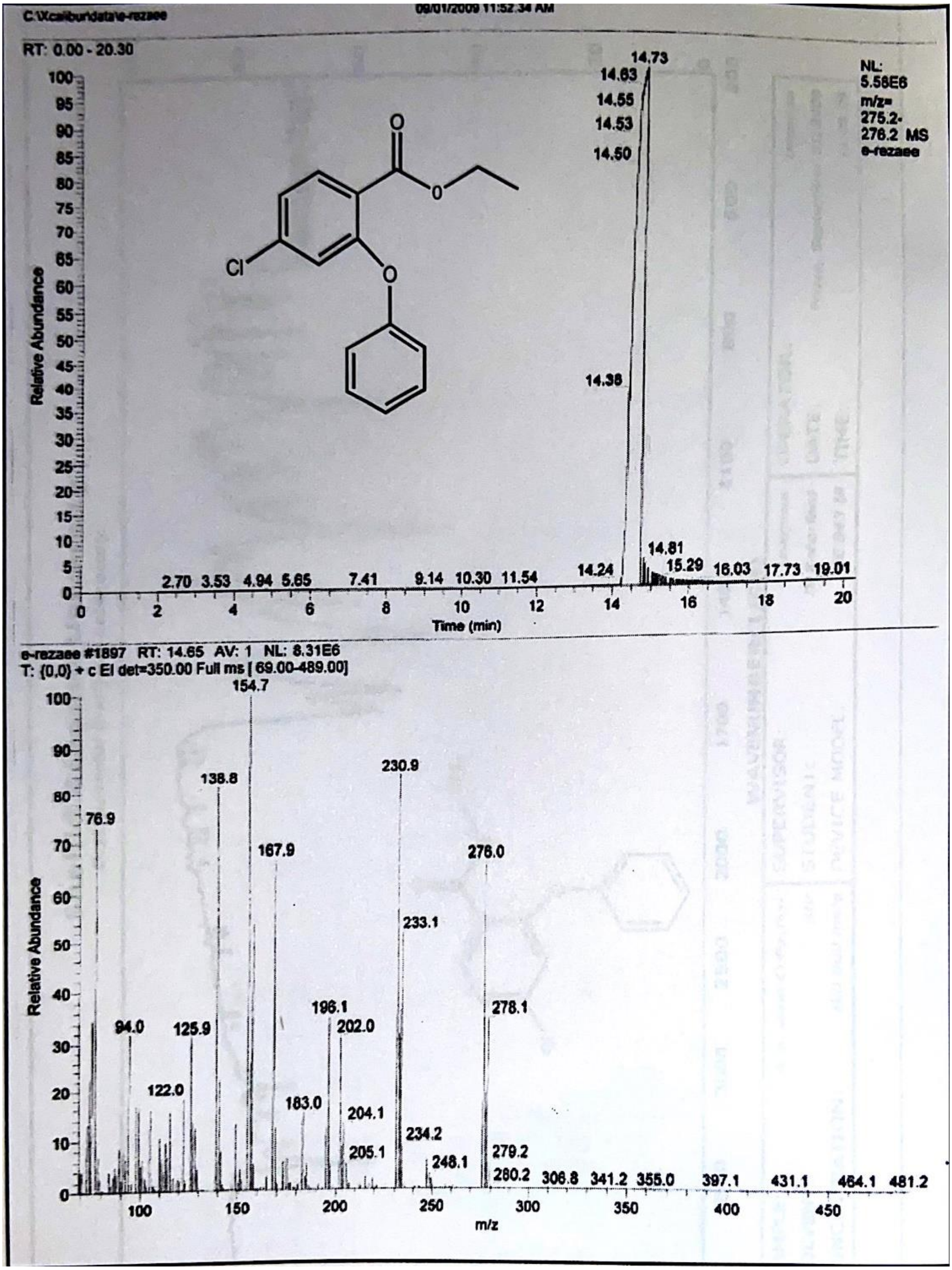

EXCLI Journal 2023;22:250-262 – ISSN 1611-2156
Received: November 28, 2022, accepted: February 02, 2023, published: February 20, 2023

Supplementary data (12): Analysis data (IR, Mass) of 4-chloro-2-phenoxybenzohydrazide (3)

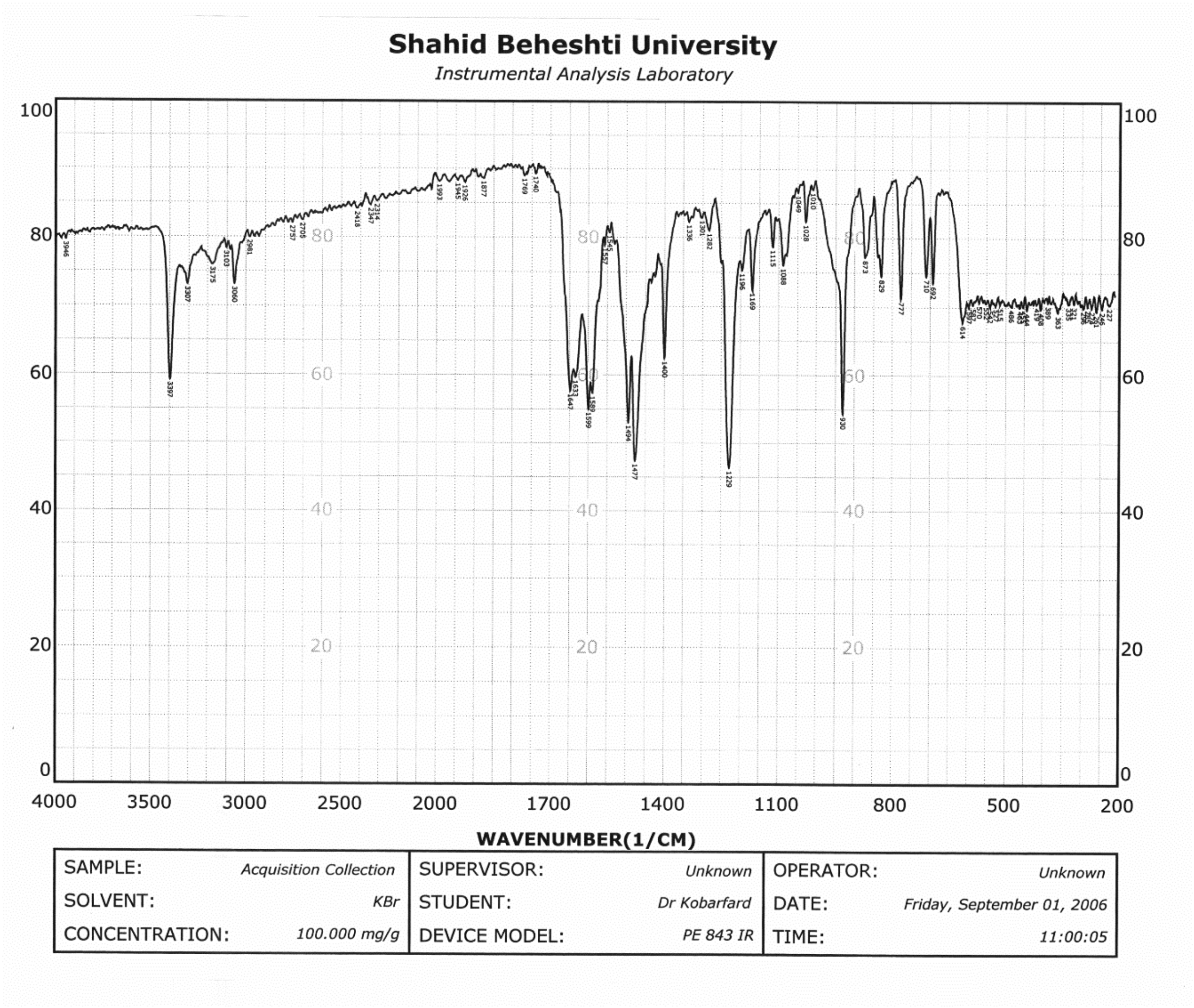

EXCLI Journal 2023;22:250-262 – ISSN 1611-2156
Received: November 28, 2022, accepted: February 02, 2023, published: February 20, 2023

Supplementary data (13): Analysis data (IR, Mass) of 4-chloro-2-phenoxybenzohydrazide (3)

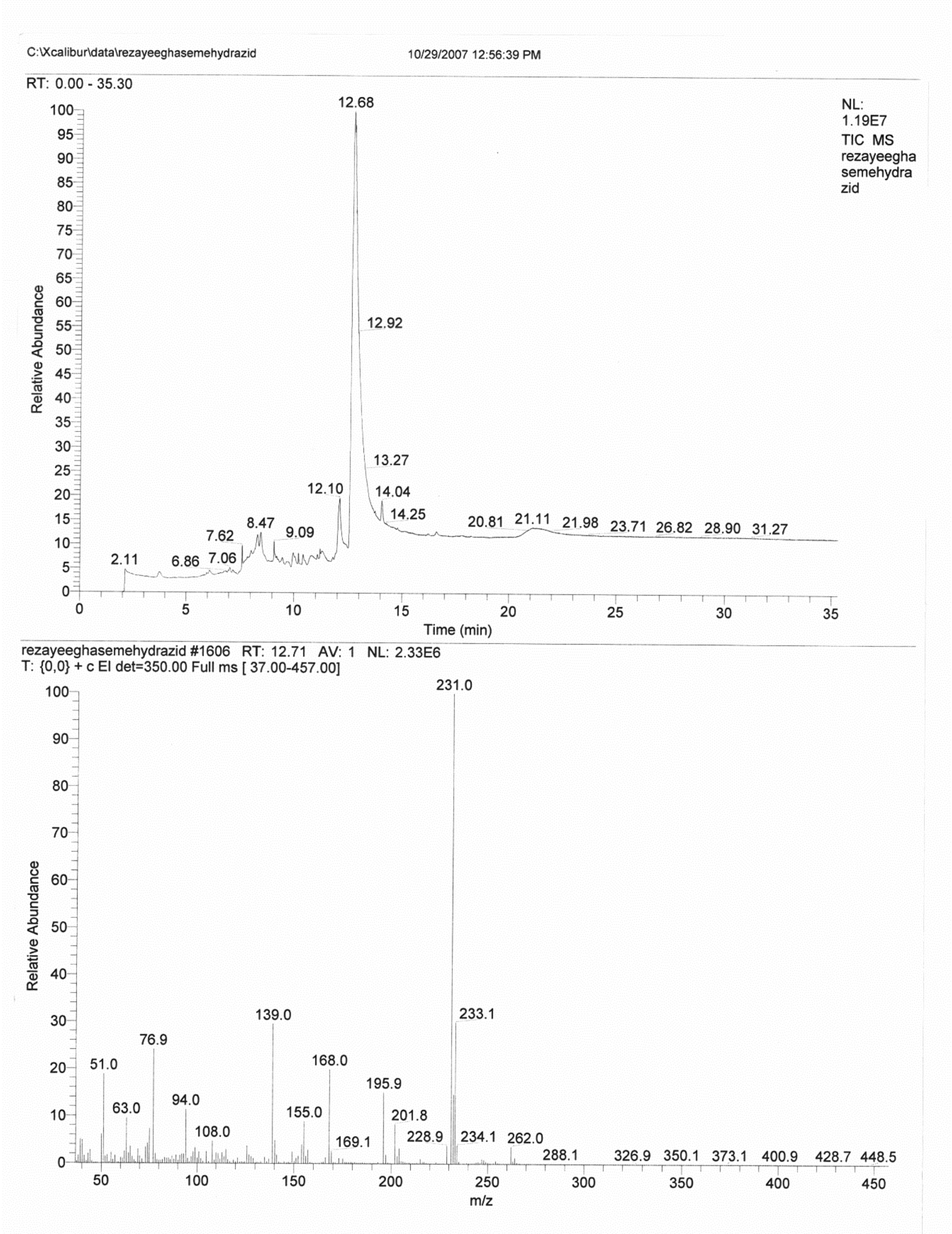

EXCLI Journal 2023;22:250-262 – ISSN 1611-2156
Received: November 28, 2022, accepted: February 02, 2023, published: February 20, 2023

Supplementary data (14): Analysis data (IR, Mass) of Ethyl 2-(2-(4-chloro-2-phenoxybenzoyl)hydrazinyl)-2-oxoacetate (4)

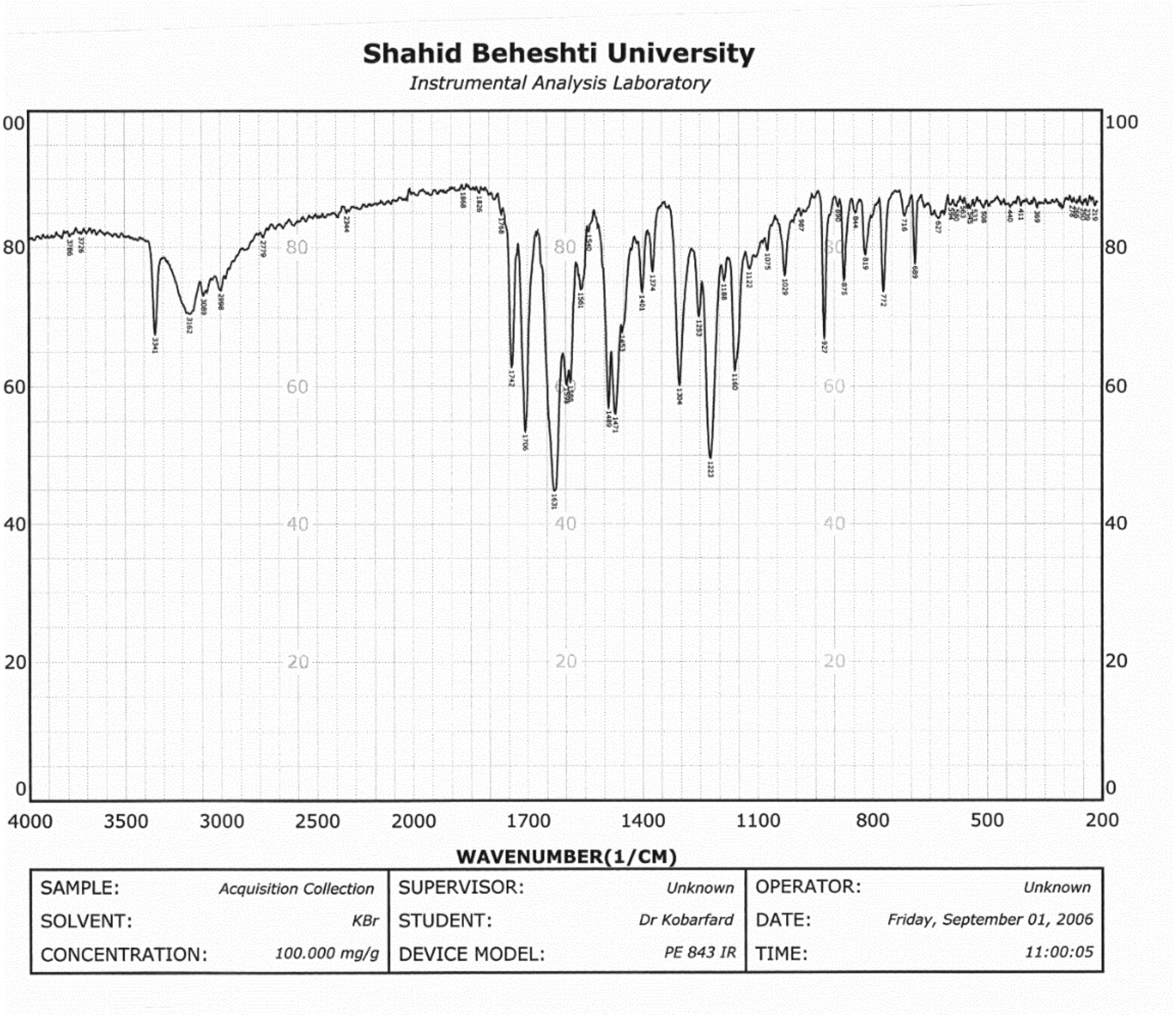

EXCLI Journal 2023;22:250-262 – ISSN 1611-2156
Received: November 28, 2022, accepted: February 02, 2023, published: February 20, 2023

Supplementary data (15): Analysis data (IR, Mass, HNMR, CNMR) of Ethyl 5-(4-chloro-2-phenoxy-phenyl)-1,3,4 oxadiazole-2- carboxylate (5)

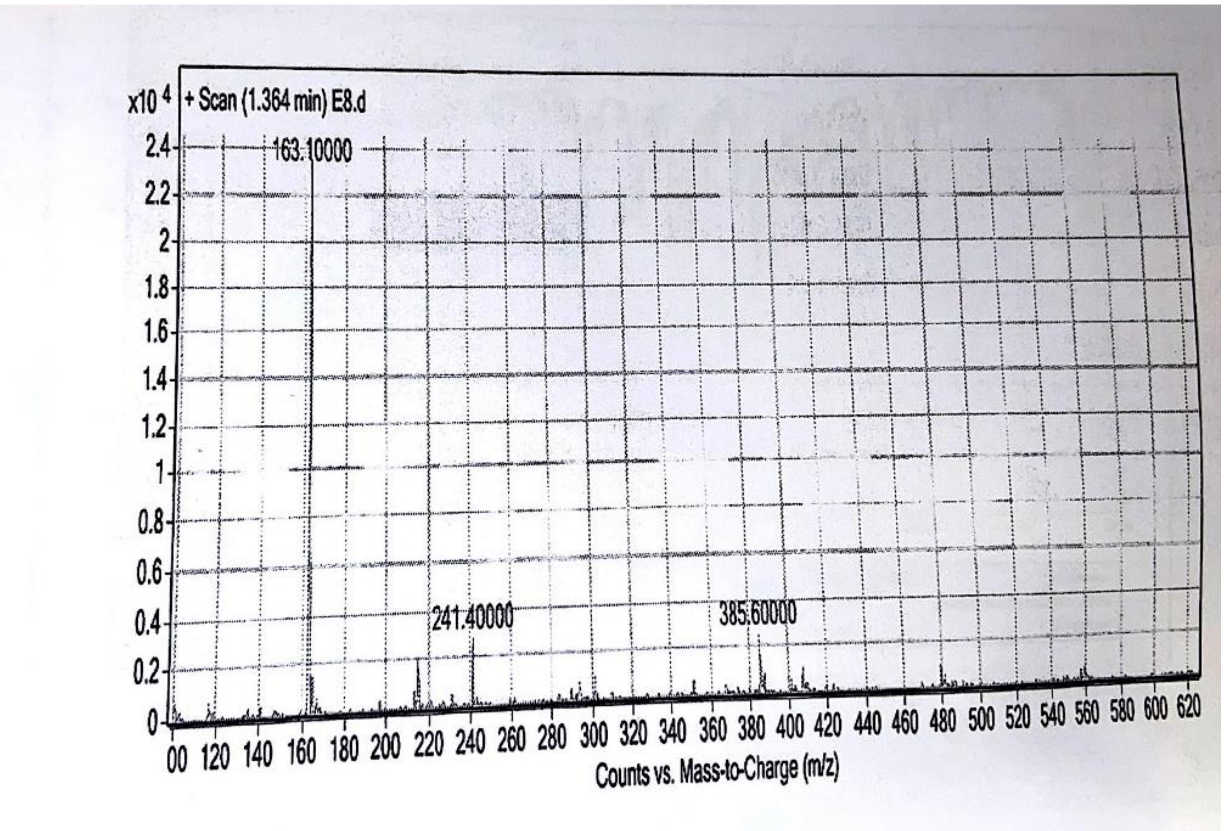

EXCLI Journal 2023;22:250-262 – ISSN 1611-2156
Received: November 28, 2022, accepted: February 02, 2023, published: February 20, 2023

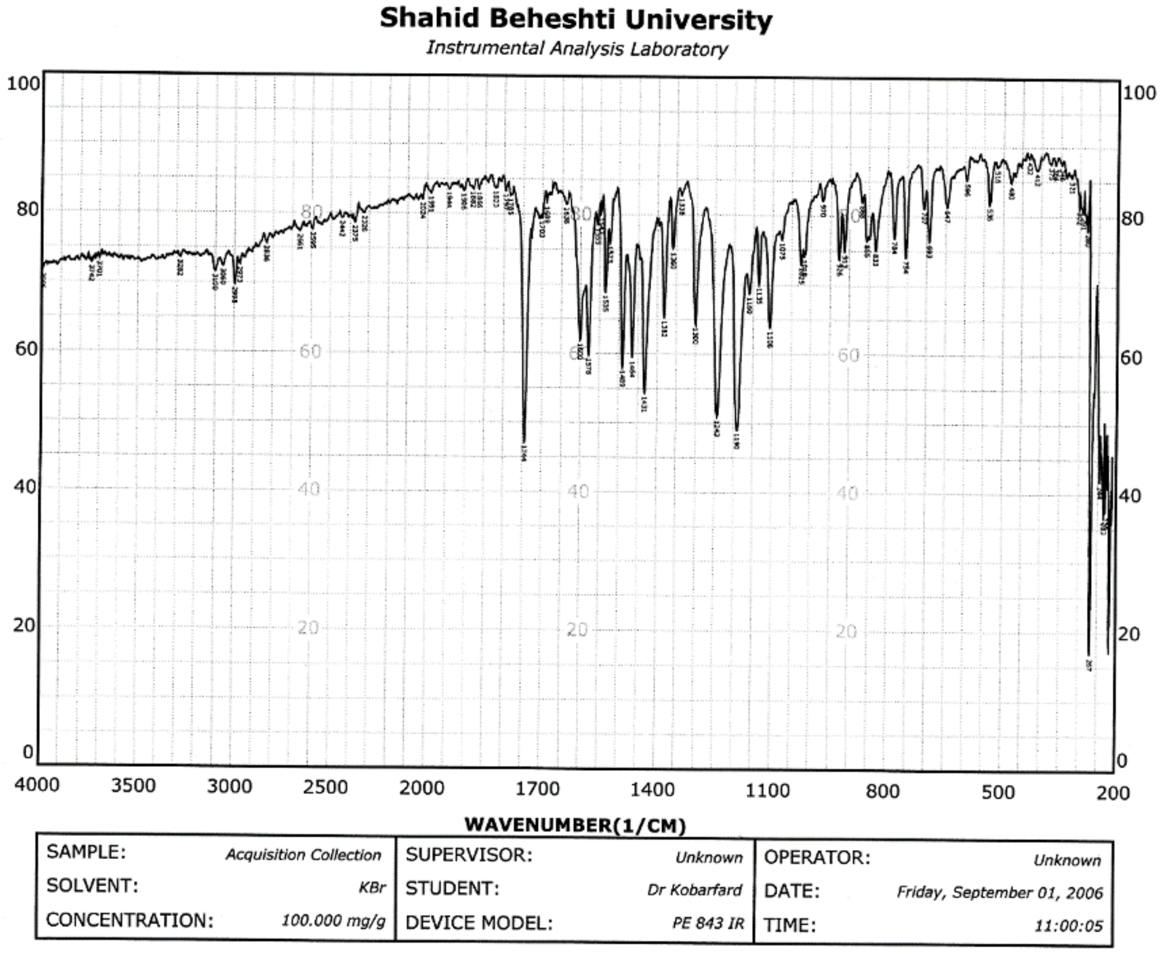

EXCLI Journal 2023;22:250-262 – ISSN 1611-2156
Received: November 28, 2022, accepted: February 02, 2023, published: February 20, 2023

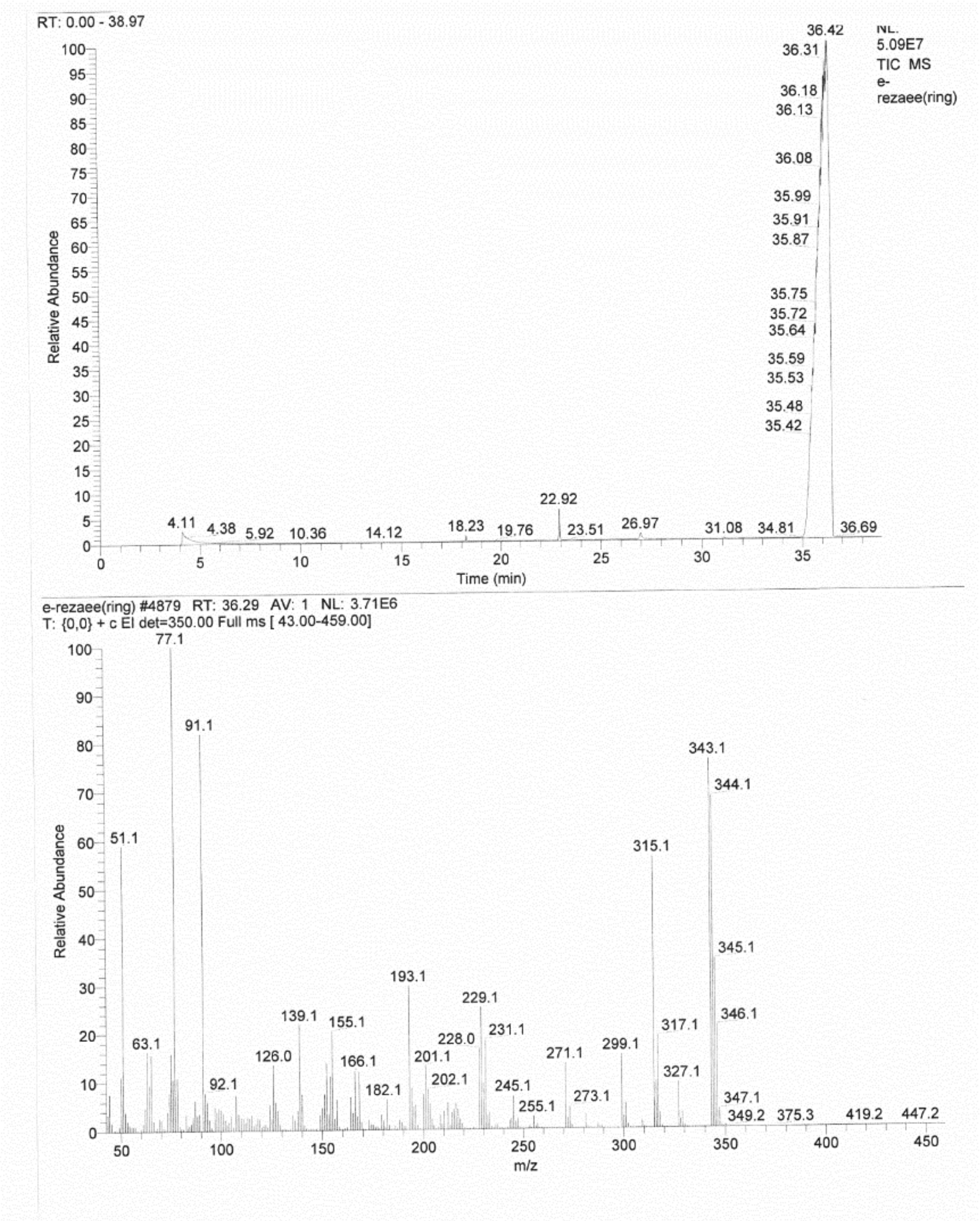

EXCLI Journal 2023;22:250-262 – ISSN 1611-2156
Received: November 28, 2022, accepted: February 02, 2023, published: February 20, 2023

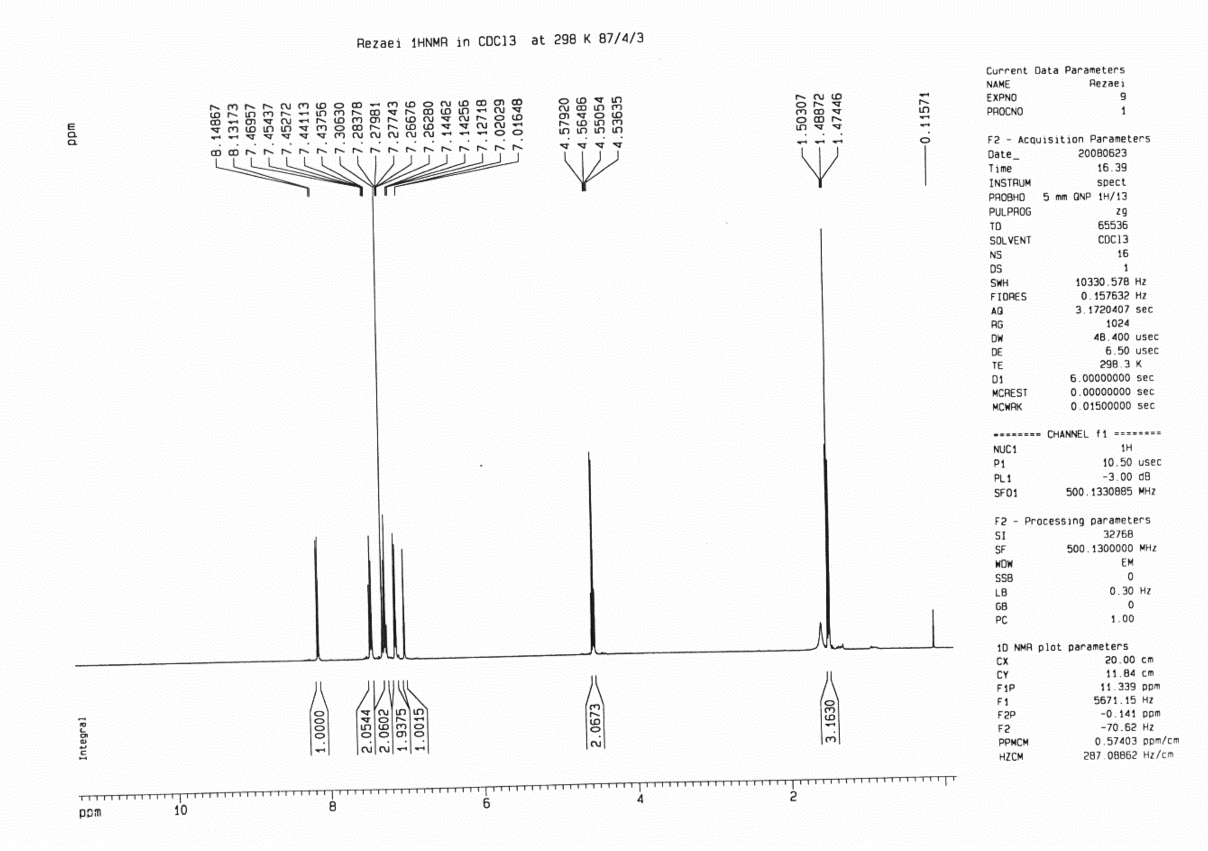

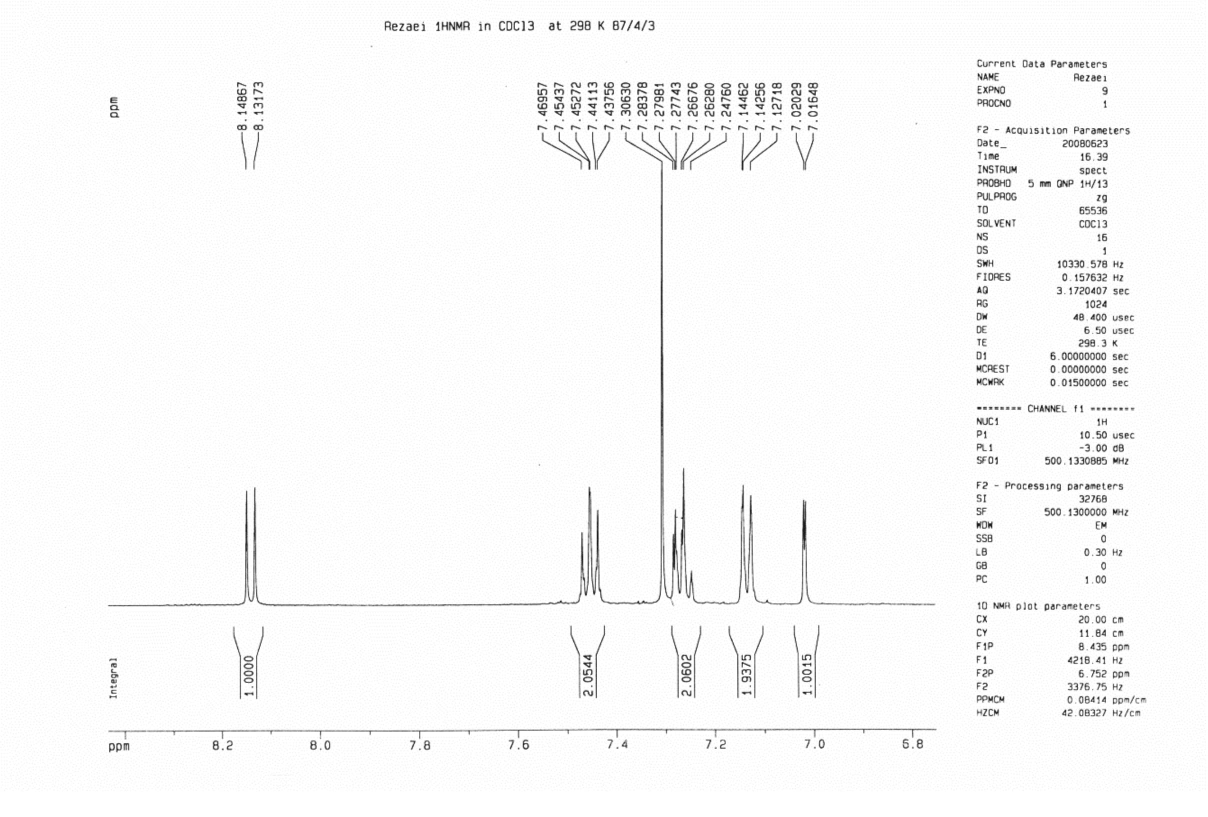

EXCLI Journal 2023;22:250-262 – ISSN 1611-2156
Received: November 28, 2022, accepted: February 02, 2023, published: February 20, 2023

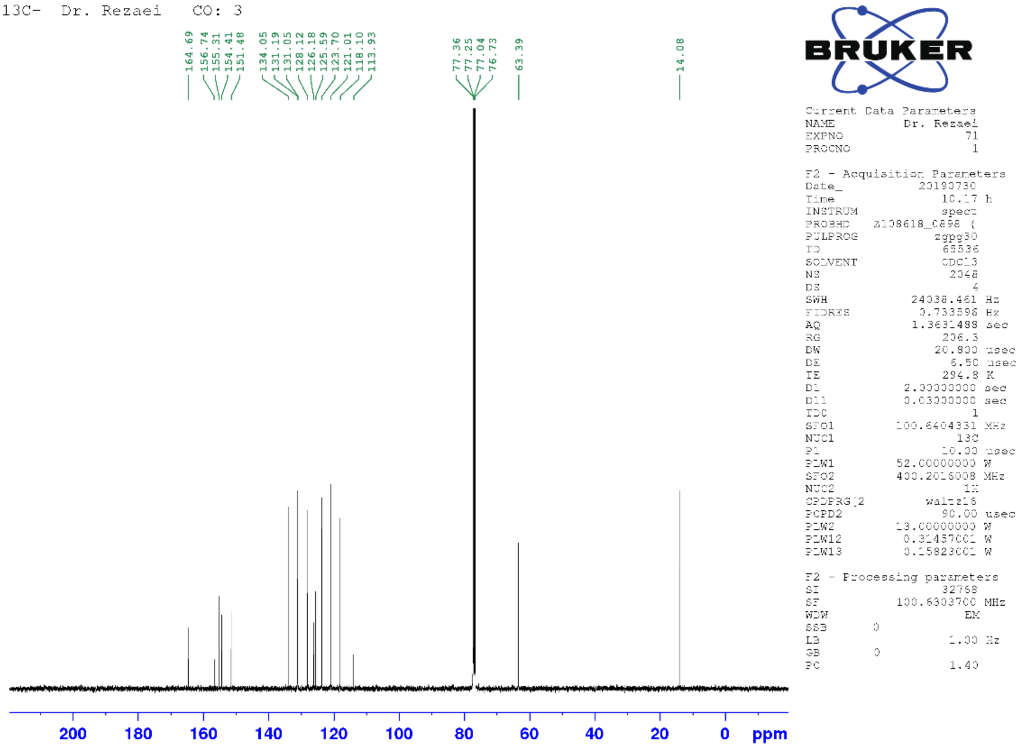

EXCLI Journal 2023;22:250-262 – ISSN 1611-2156
Received: November 28, 2022, accepted: February 02, 2023, published: February 20, 2023

Supplementary data (16): Analysis data (IR, Mass, ^{H}NMR, ^{C}NMR) of 5-(4-chloro-2-phenoxyphenyl)-N-phenyl-1,3,4-oxadiazole-2-Carboxamide (6a)

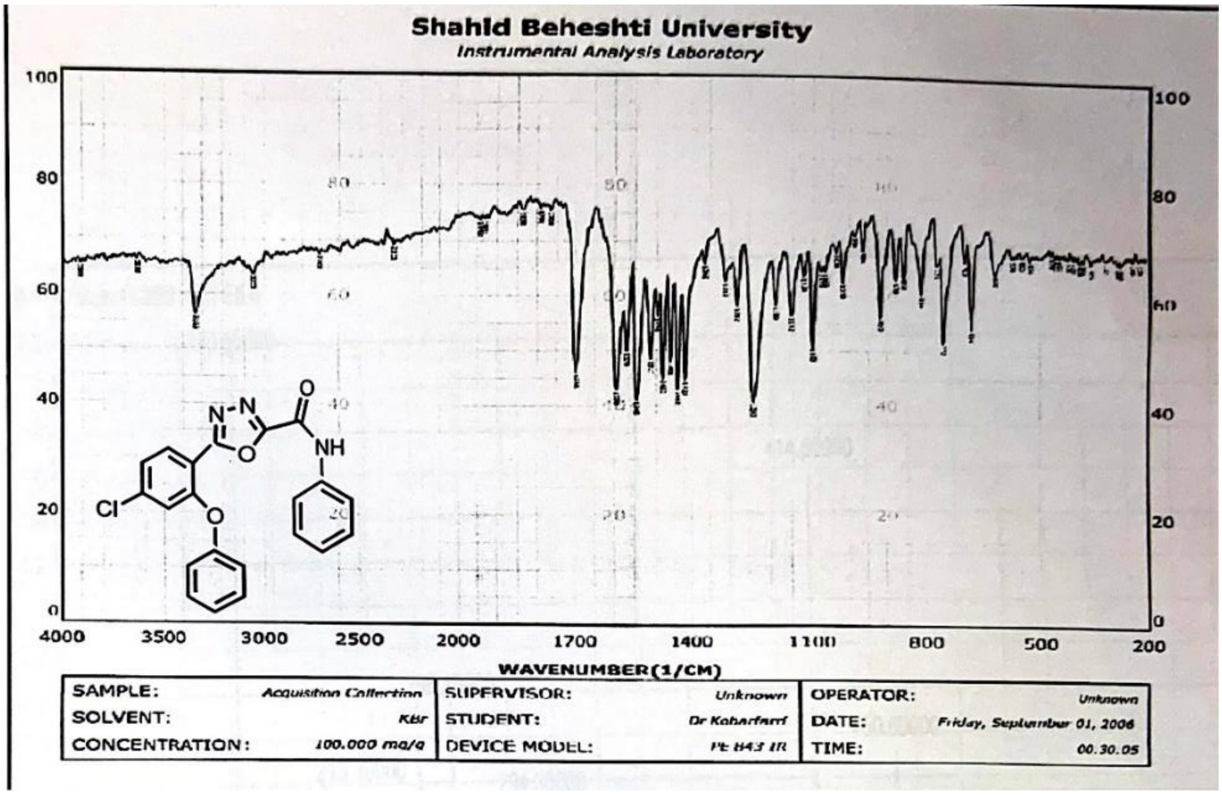

EXCLI Journal 2023;22:250-262 – ISSN 1611-2156
Received: November 28, 2022, accepted: February 02, 2023, published: February 20, 2023

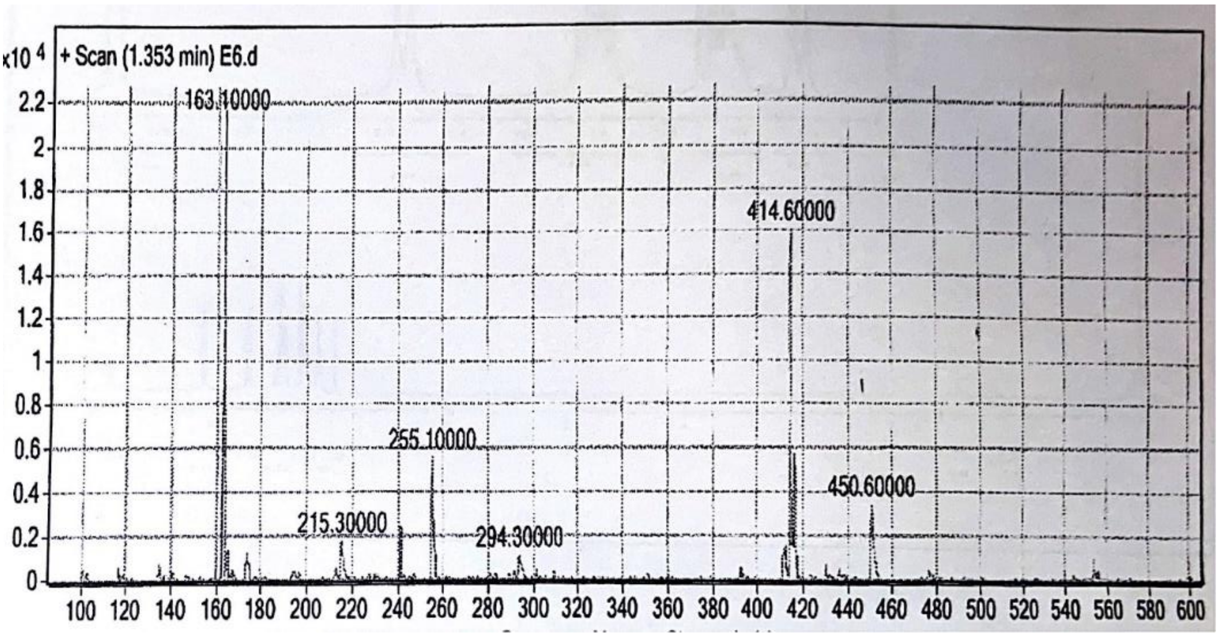

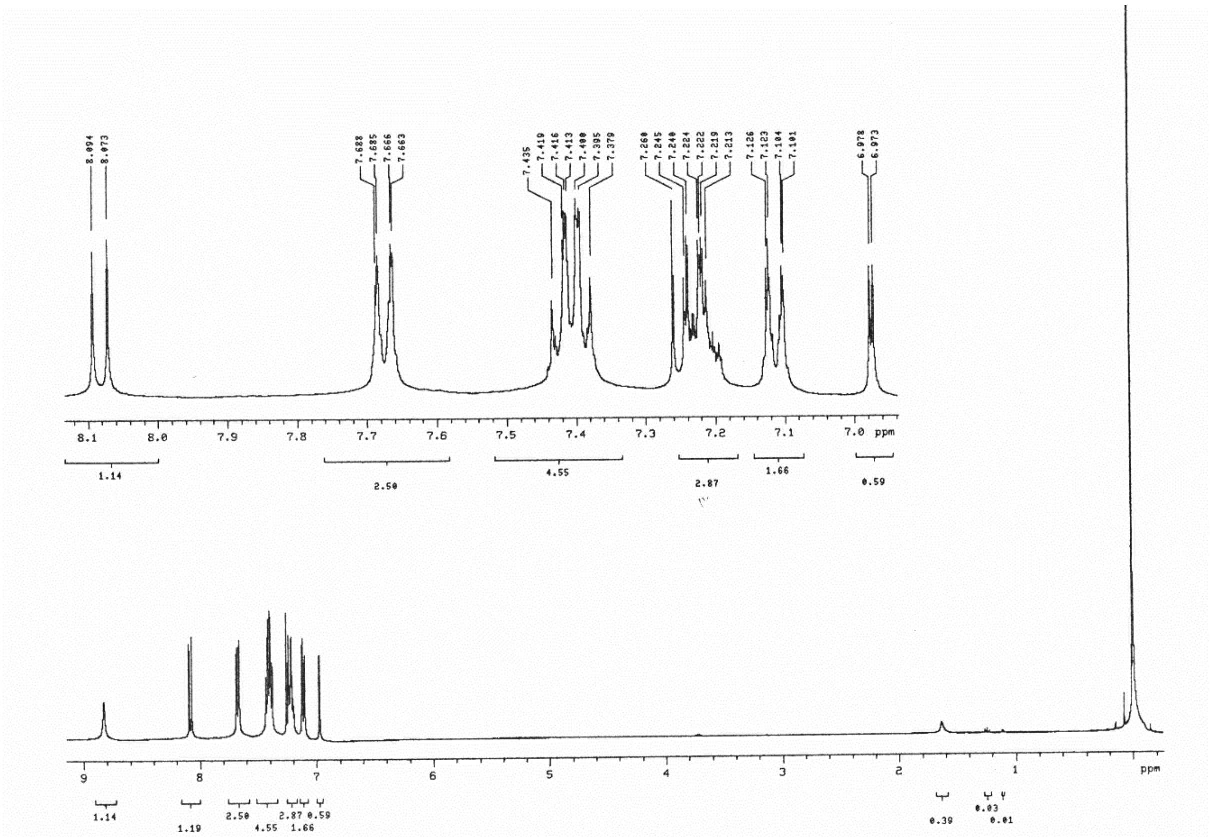

EXCLI Journal 2023;22:250-262 – ISSN 1611-2156
Received: November 28, 2022, accepted: February 02, 2023, published: February 20, 2023

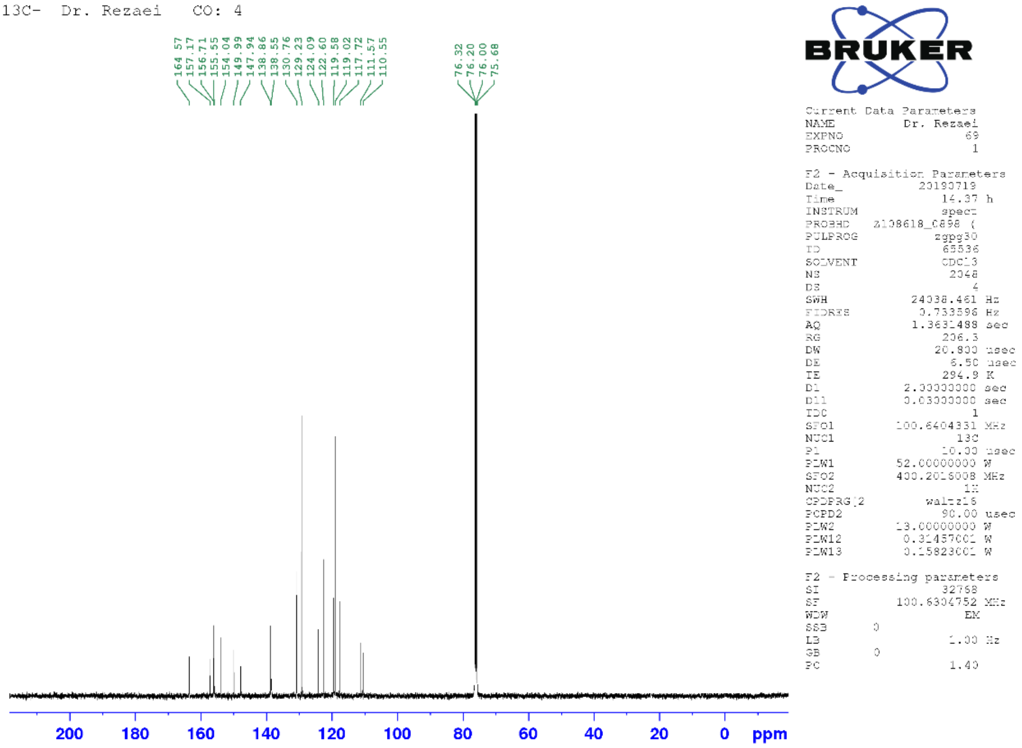

EXCLI Journal 2023;22:250-262 – ISSN 1611-2156
Received: November 28, 2022, accepted: February 02, 2023, published: February 20, 2023

Supplementary data (17): Analysis data (IR, Mass, HNMR, CNMR) of 5-(4-chloro-2-phenoxyphenyl)-N-(pyridin-2-yl)-1,3,4-oxadiazole-2-Carboxamide (6b)

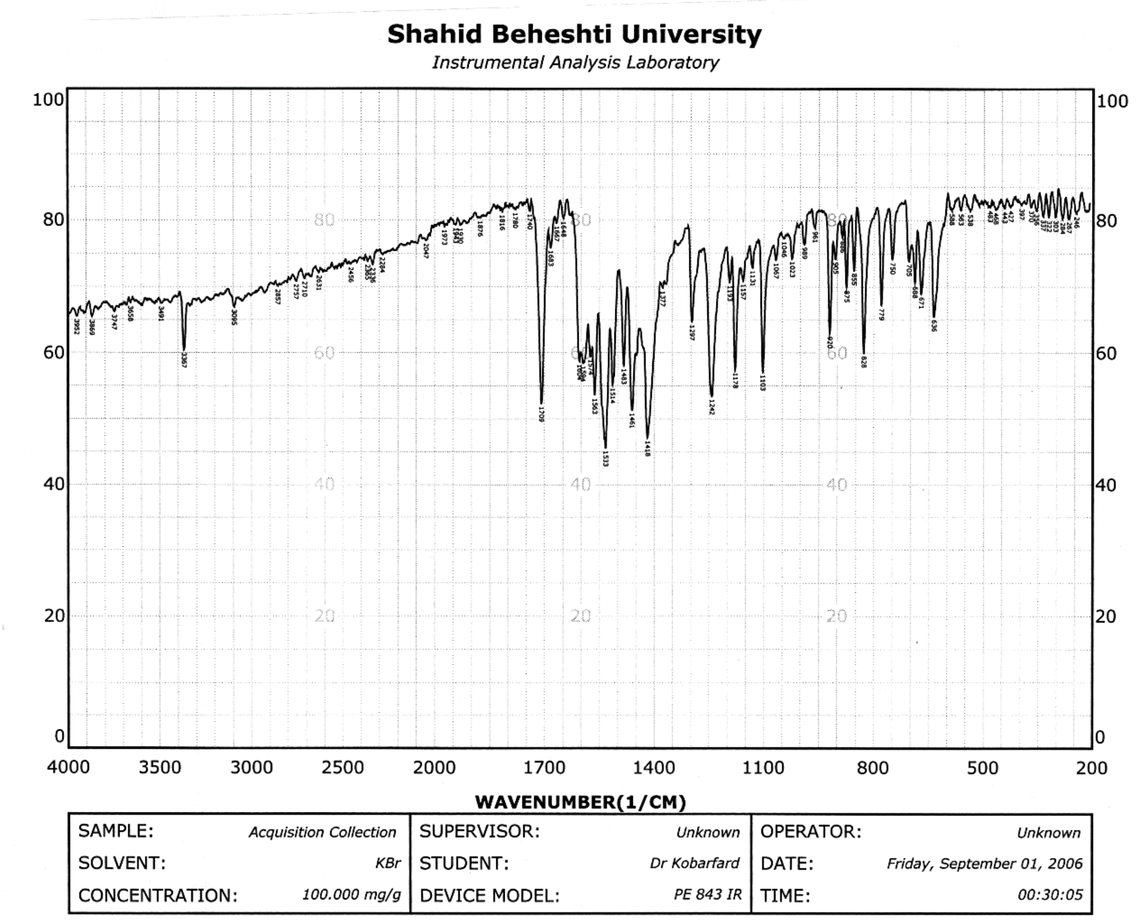

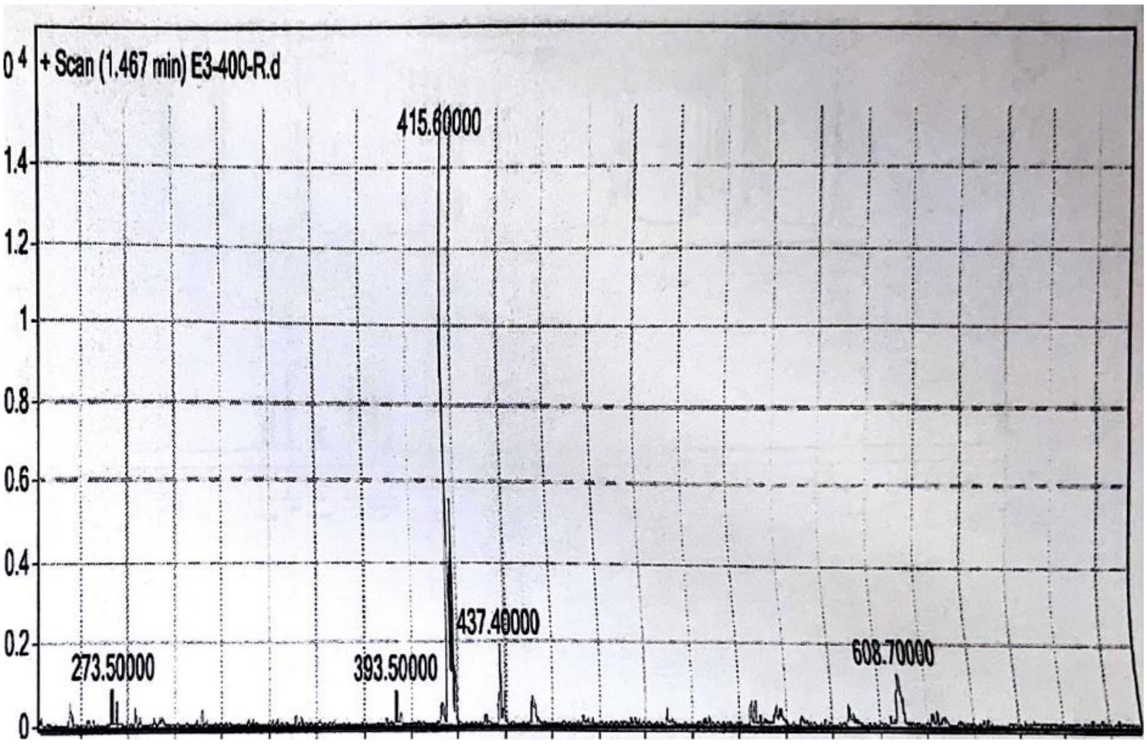

EXCLI Journal 2023;22:250-262 – ISSN 1611-2156
Received: November 28, 2022, accepted: February 02, 2023, published: February 20, 2023

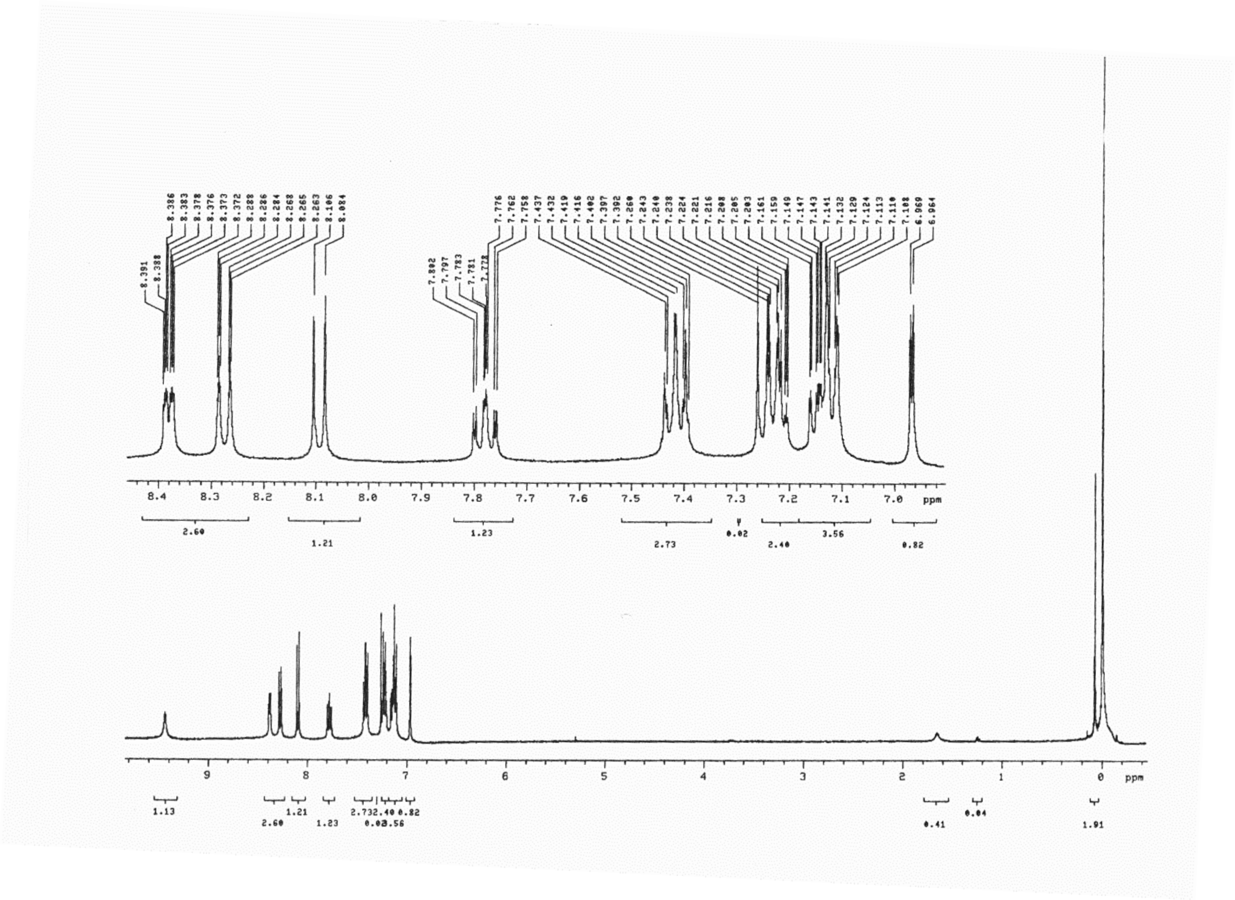

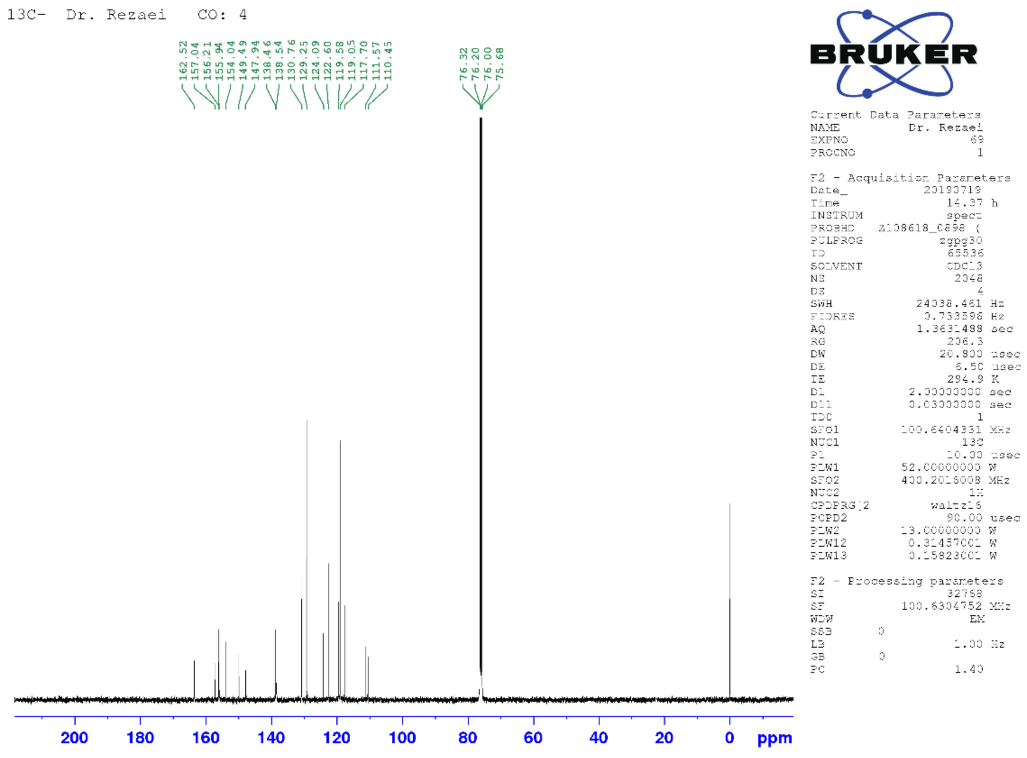

EXCLI Journal 2023;22:250-262 – ISSN 1611-2156
Received: November 28, 2022, accepted: February 02, 2023, published: February 20, 2023

Supplementary data (18): Analysis data (IR, Mass, HNMR, CNMR) of 5-(4-chloro-2-phenoxyphenyl)-N-(6-methylpyridin-2-yl)-1,3,4-oxadiazole-2-Carboxamide (6c)

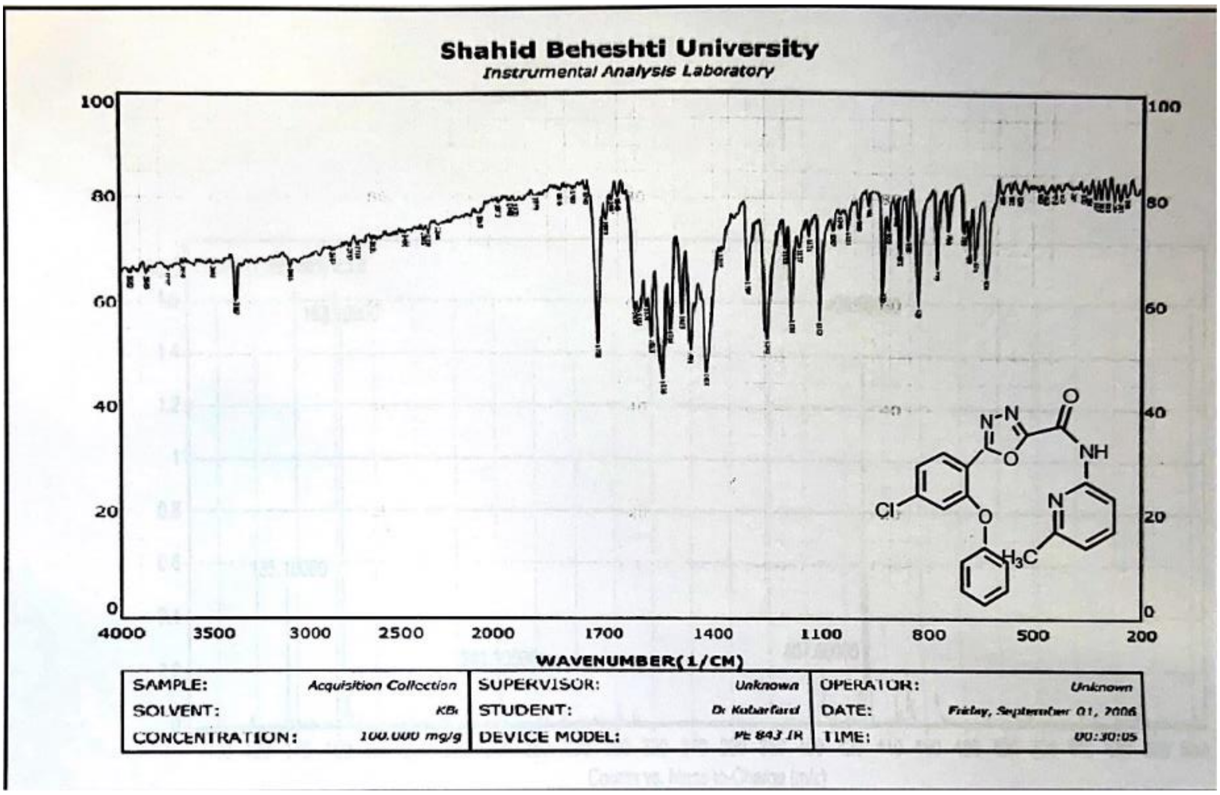

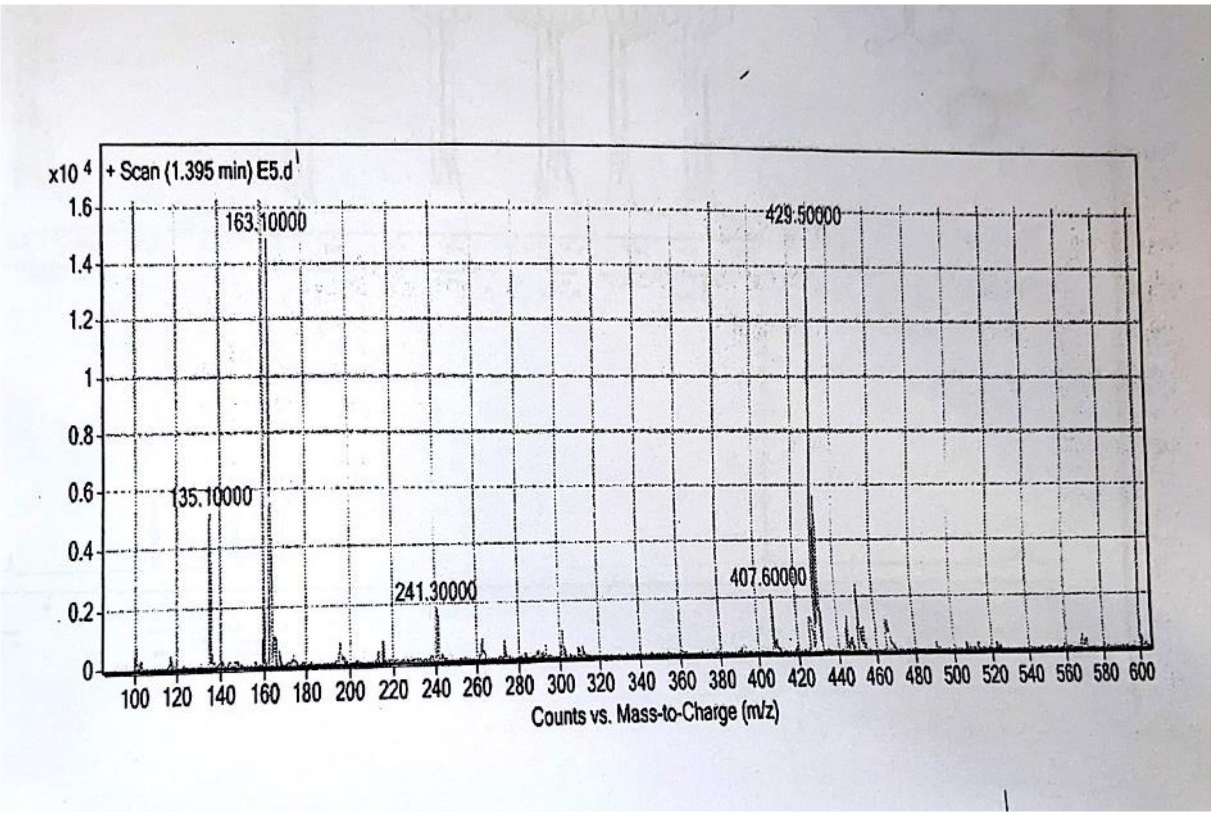

EXCLI Journal 2023;22:250-262 – ISSN 1611-2156
Received: November 28, 2022, accepted: February 02, 2023, published: February 20, 2023

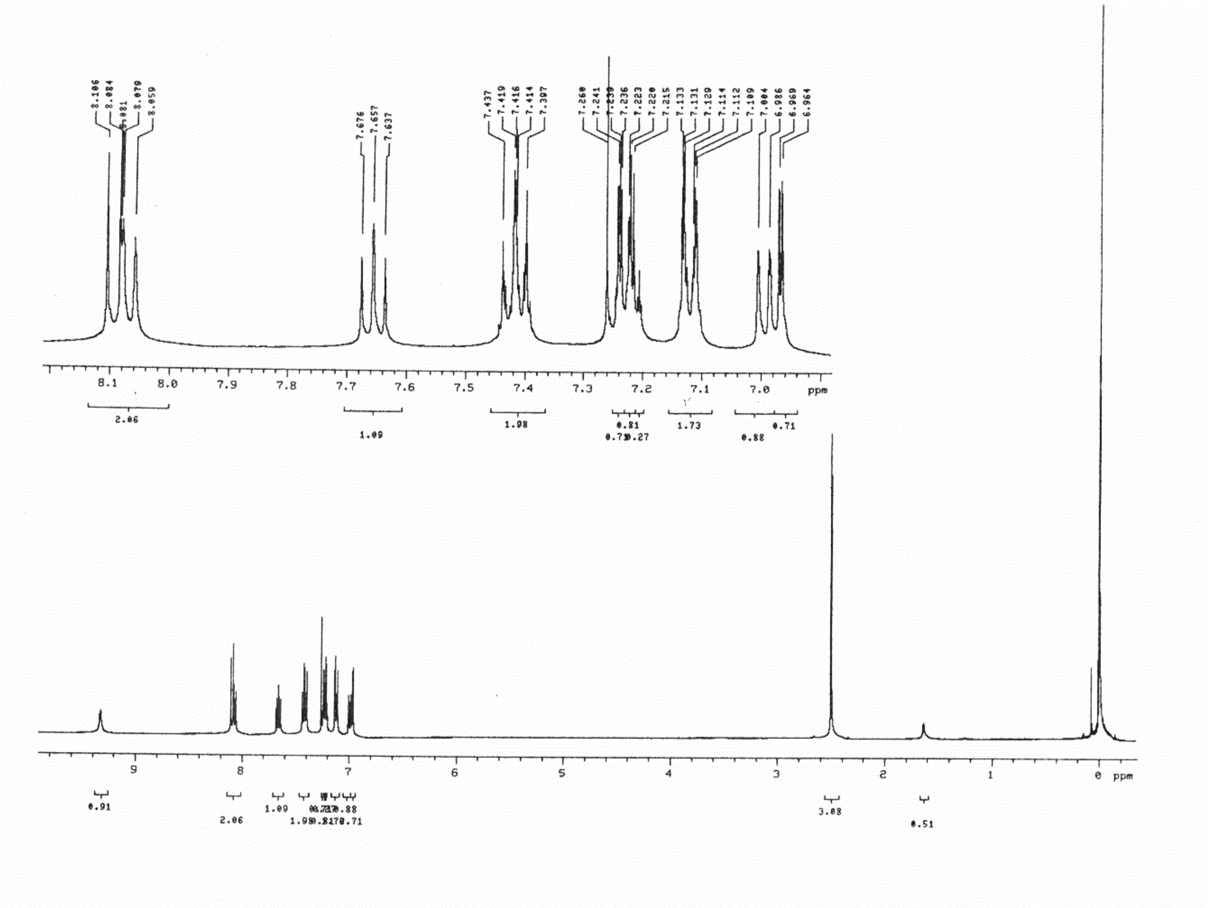

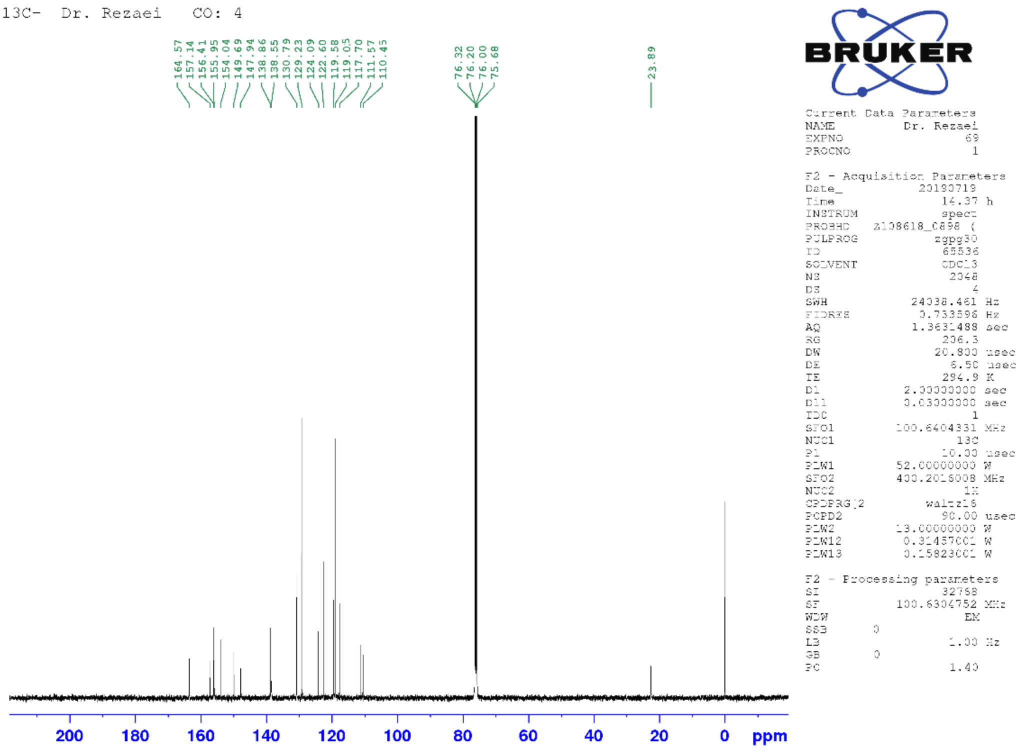

EXCLI Journal 2023;22:250-262 – ISSN 1611-2156
Received: November 28, 2022, accepted: February 02, 2023, published: February 20, 2023

Supplementary data (19): Analysis data (IR, Mass, HNMR, CNMR) of 5-(4-chloro-2-phenoxyphenyl)-N-(4-methylpyridin-2-yl)-1,3,4-oxadiazole-2-Carboxamide (6d)

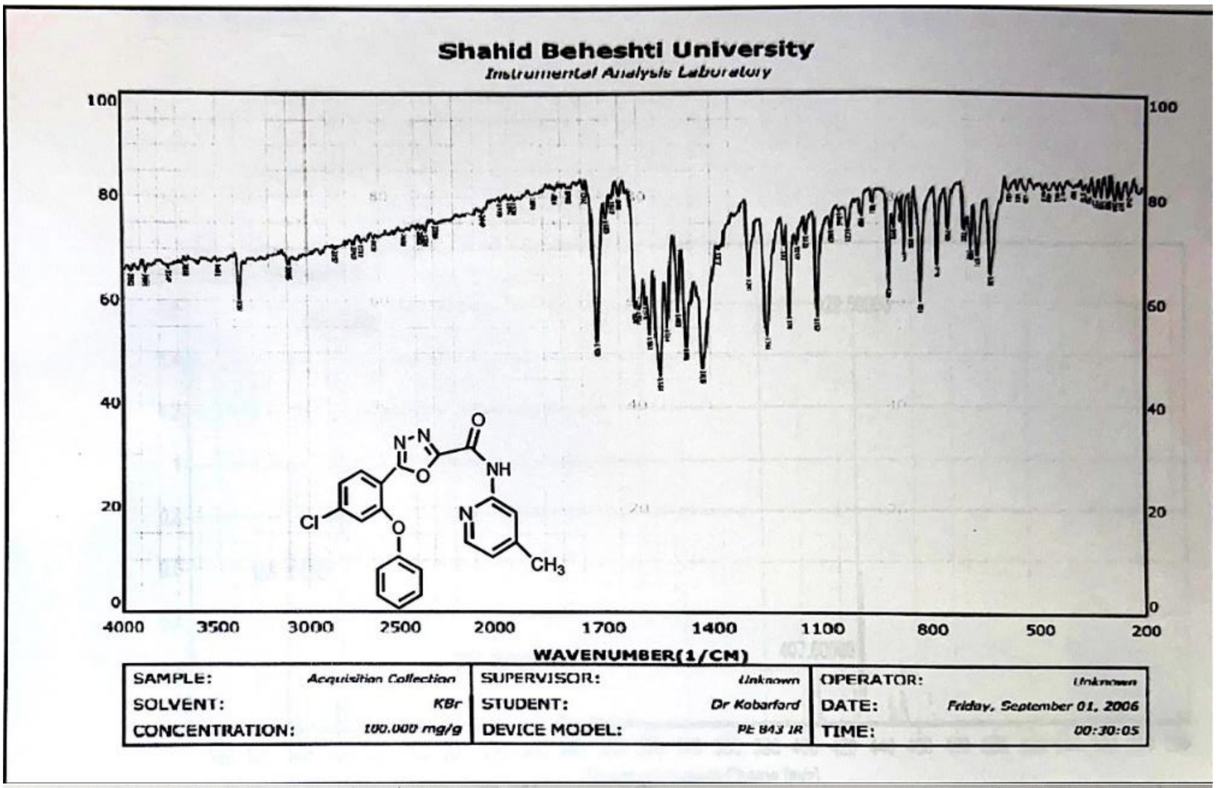

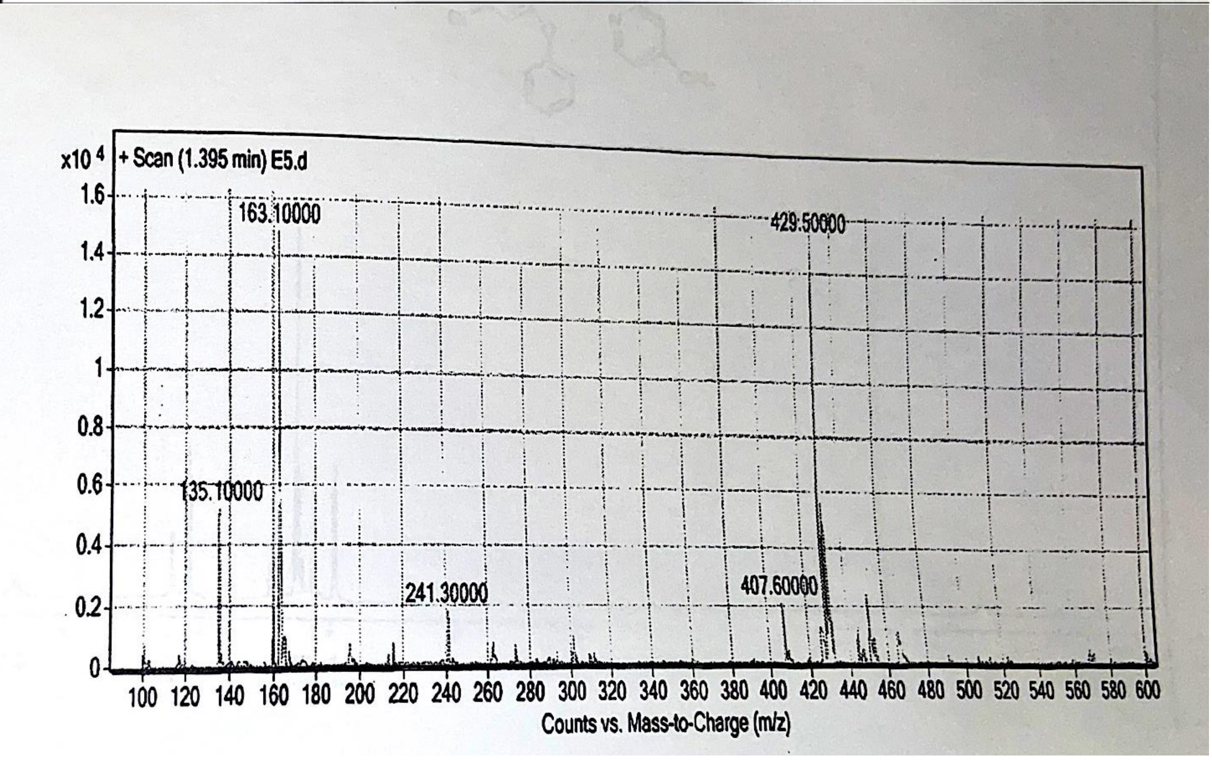

EXCLI Journal 2023;22:250-262 – ISSN 1611-2156
Received: November 28, 2022, accepted: February 02, 2023, published: February 20, 2023

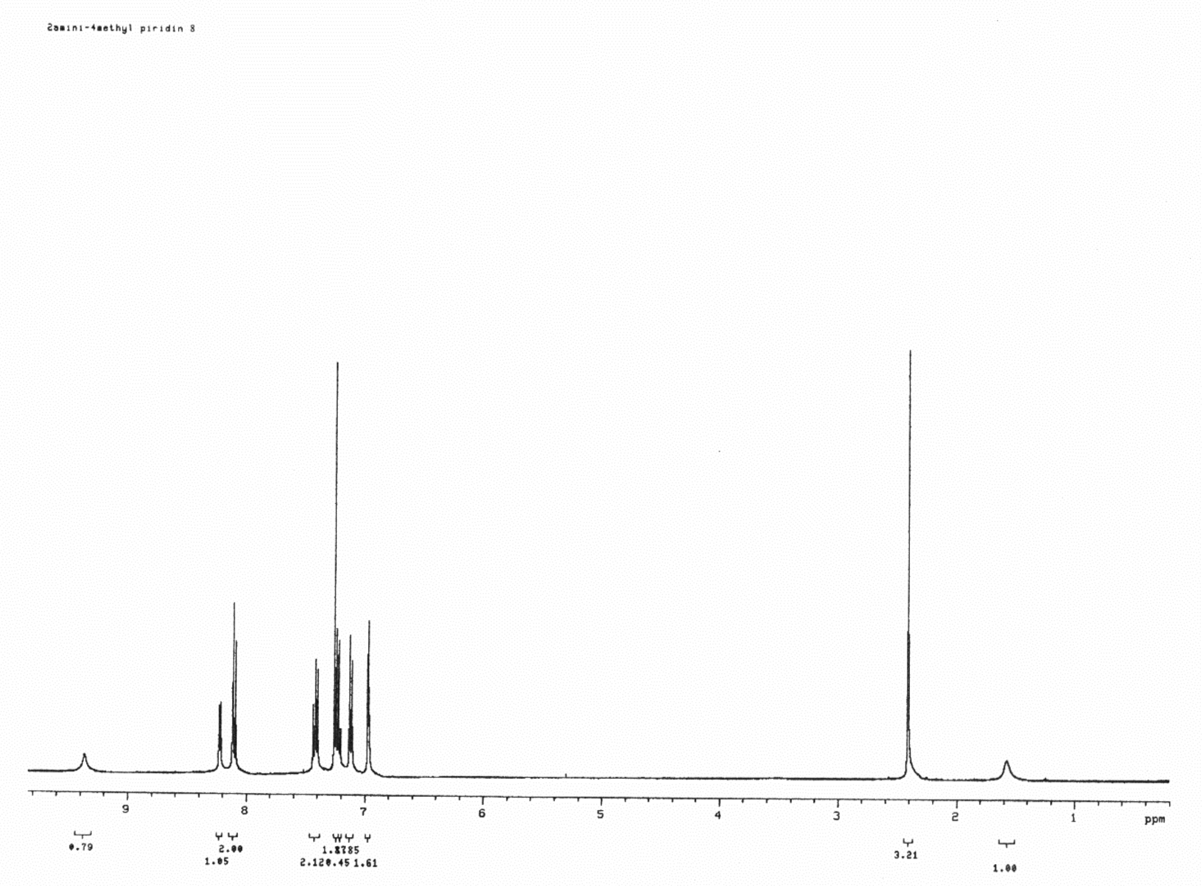

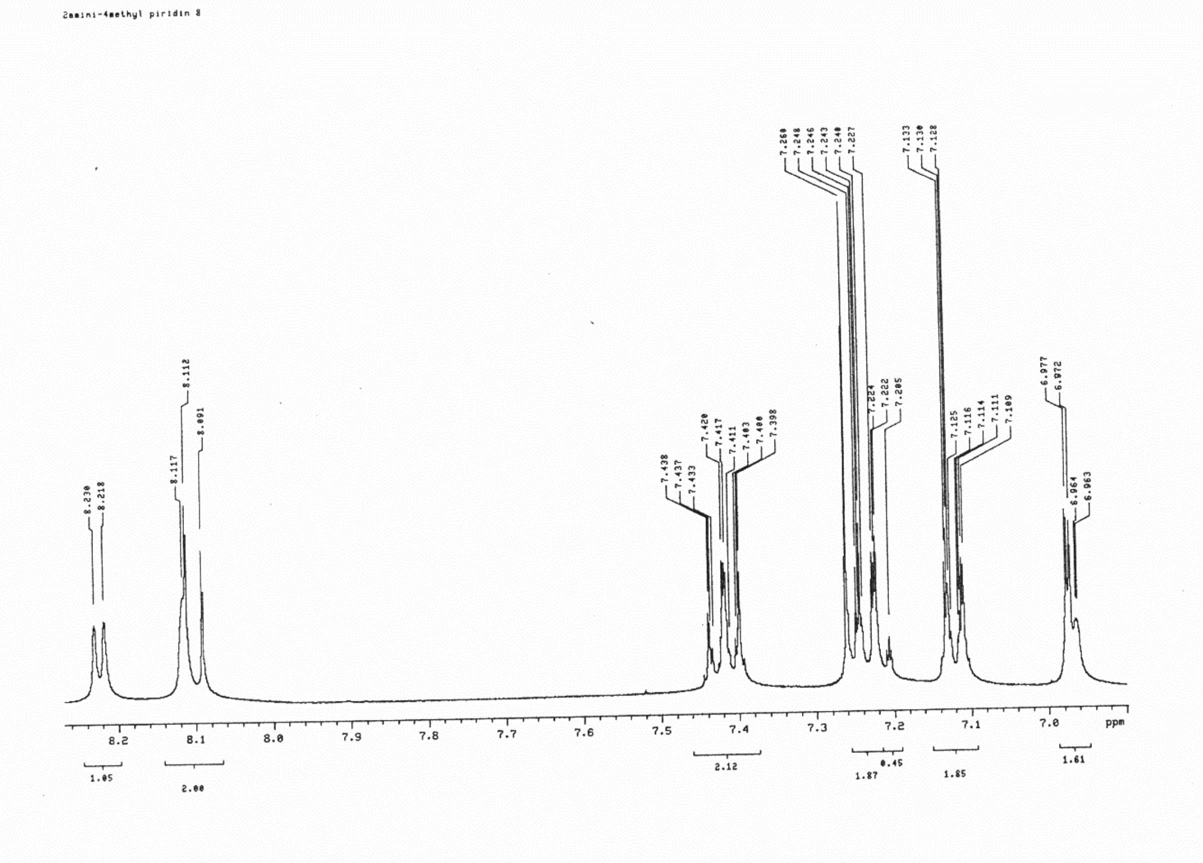

EXCLI Journal 2023;22:250-262 – ISSN 1611-2156
Received: November 28, 2022, accepted: February 02, 2023, published: February 20, 2023

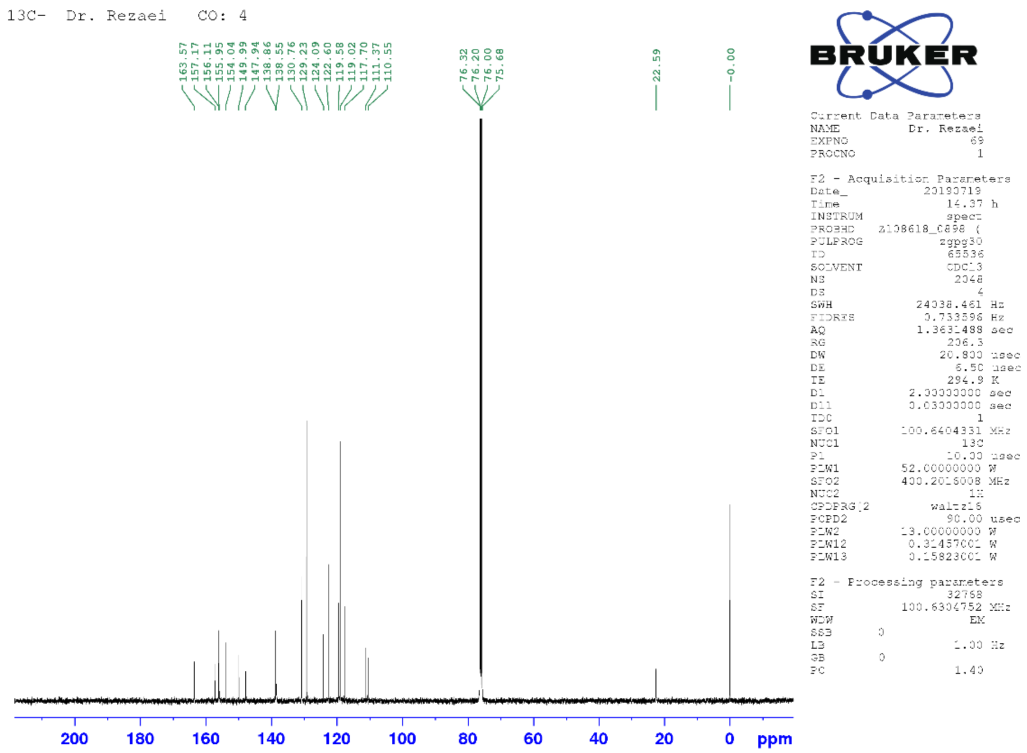

EXCLI Journal 2023;22:250-262 – ISSN 1611-2156
Received: November 28, 2022, accepted: February 02, 2023, published: February 20, 2023

Supplementary data (20): Analysis data (IR, Mass, HNMR, CNMR) of 5-(4-chloro-2-phenoxyphenyl)-N-morpholino-1,3,4-oxadiazole-2-Carboxamide (6e)

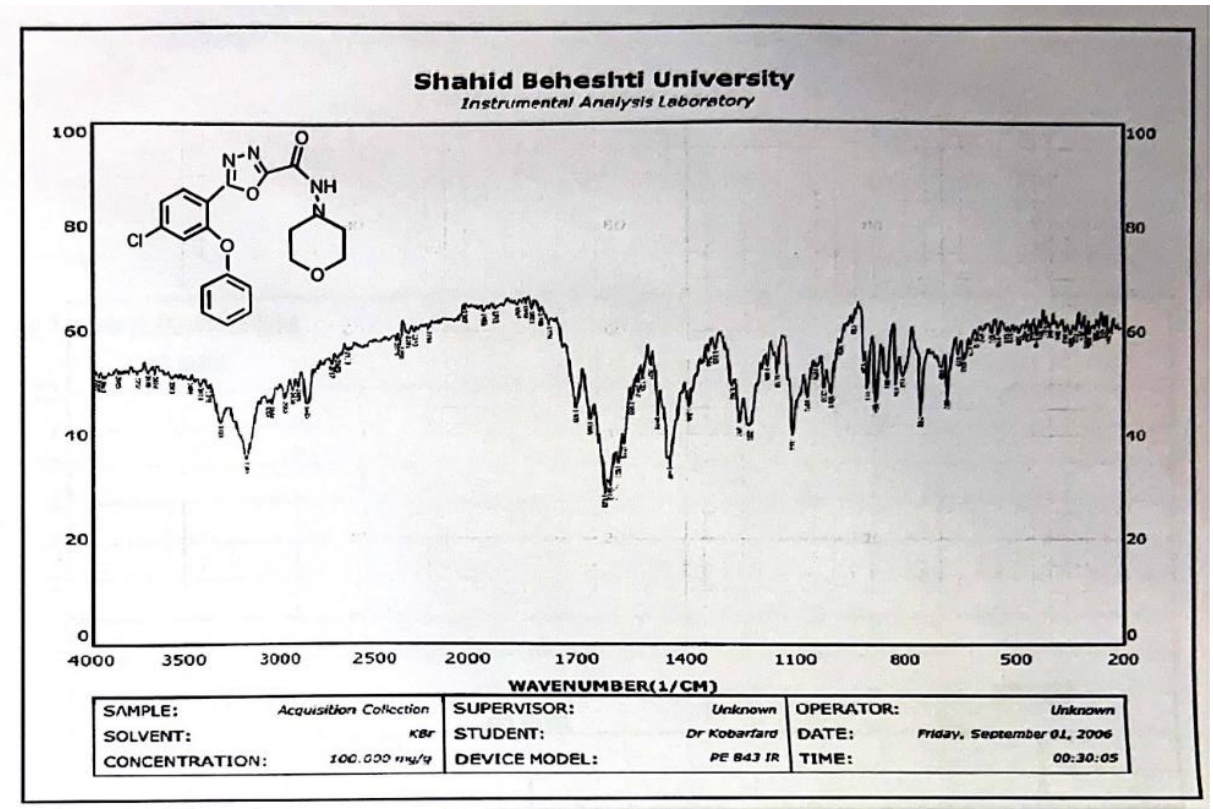

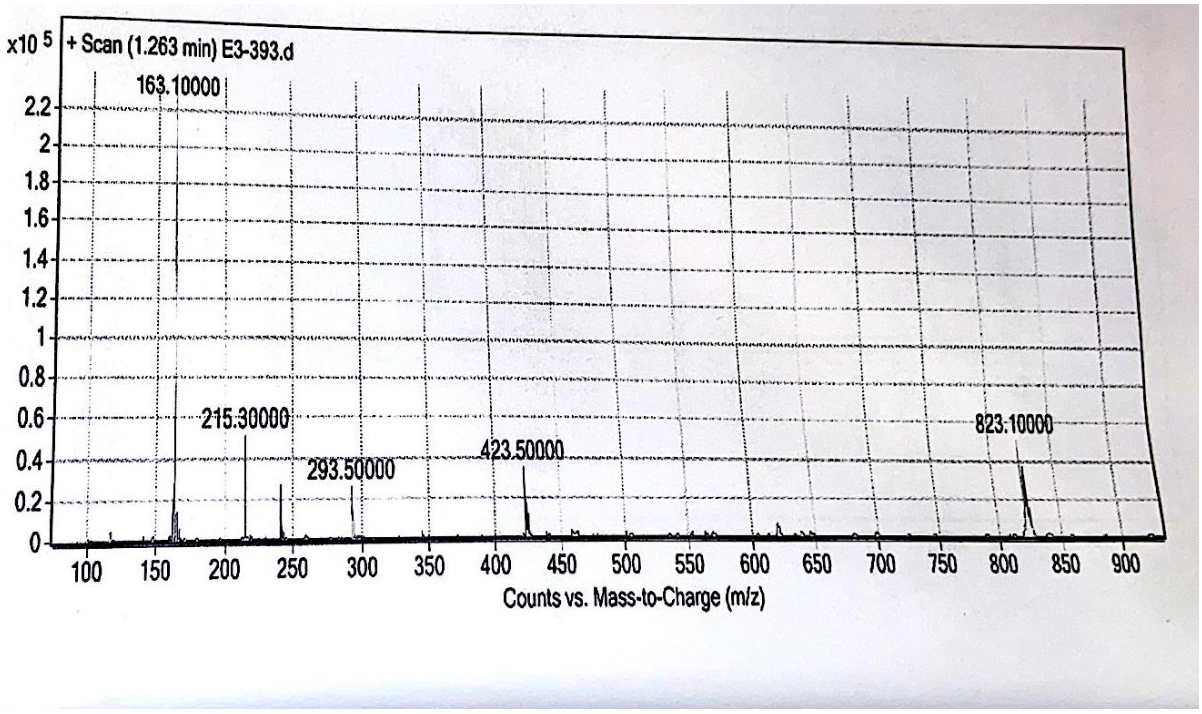

EXCLI Journal 2023;22:250-262 – ISSN 1611-2156
Received: November 28, 2022, accepted: February 02, 2023, published: February 20, 2023

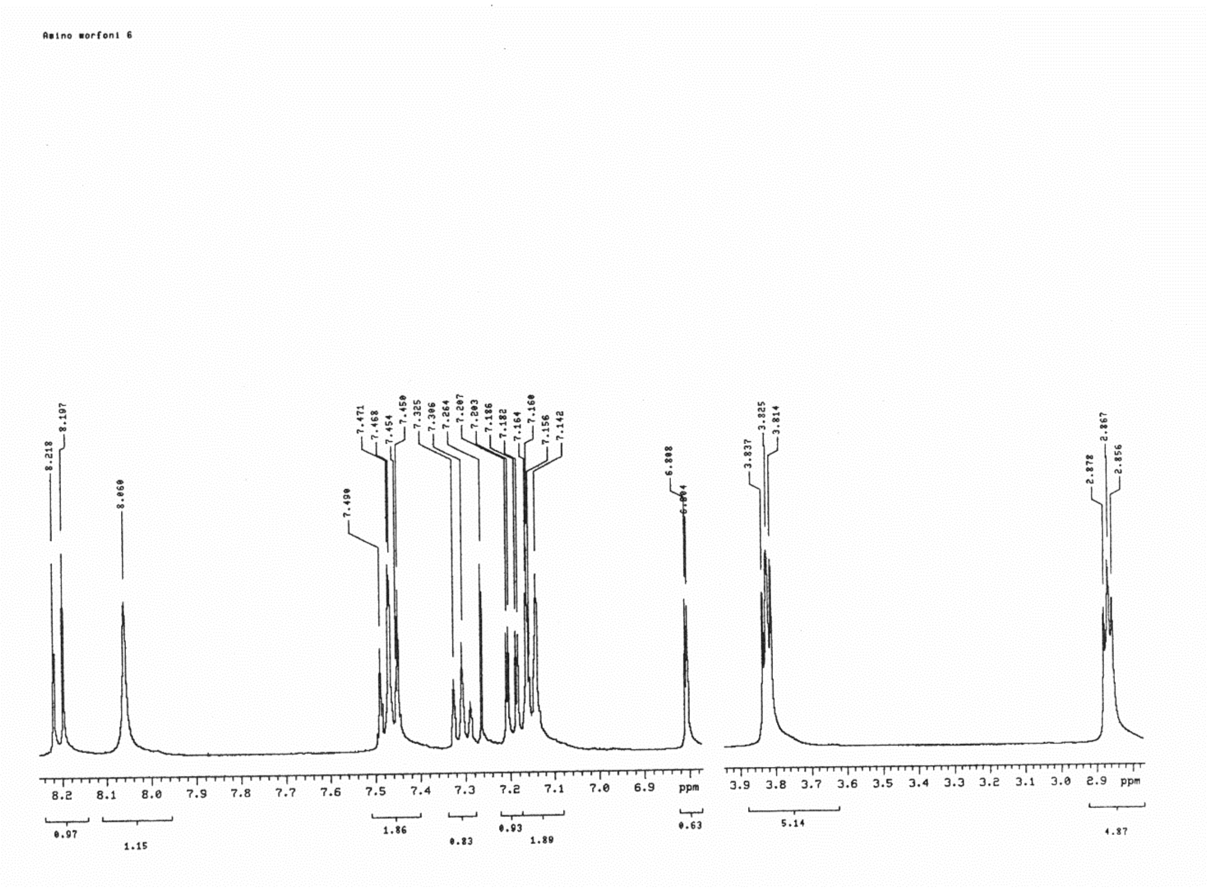

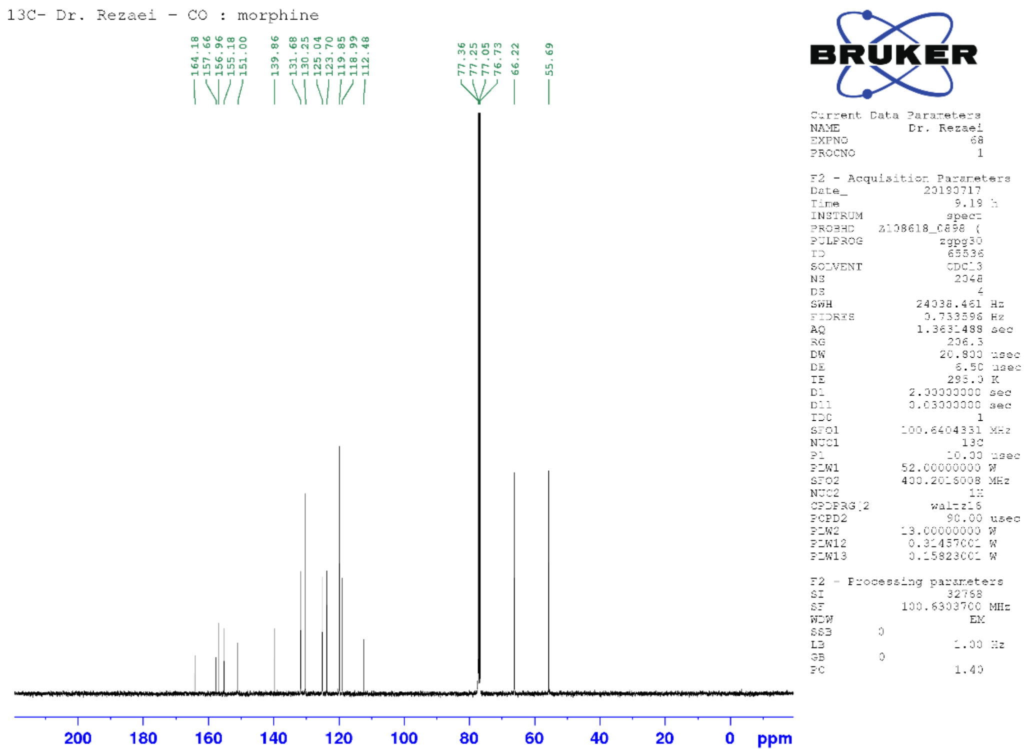

Received: November 28, 2022, accepted: February 02, 2023, published: February 20, 2023

Supplementary data (21): Analysis data (IR, Mass, ^{H}NMR, ^{C}NMR) of 5-(4-chloro-2-phenoxyphenyl)-N-cyclohexyl-1,3,4-oxadiazole-2-Carboxamide (6f)

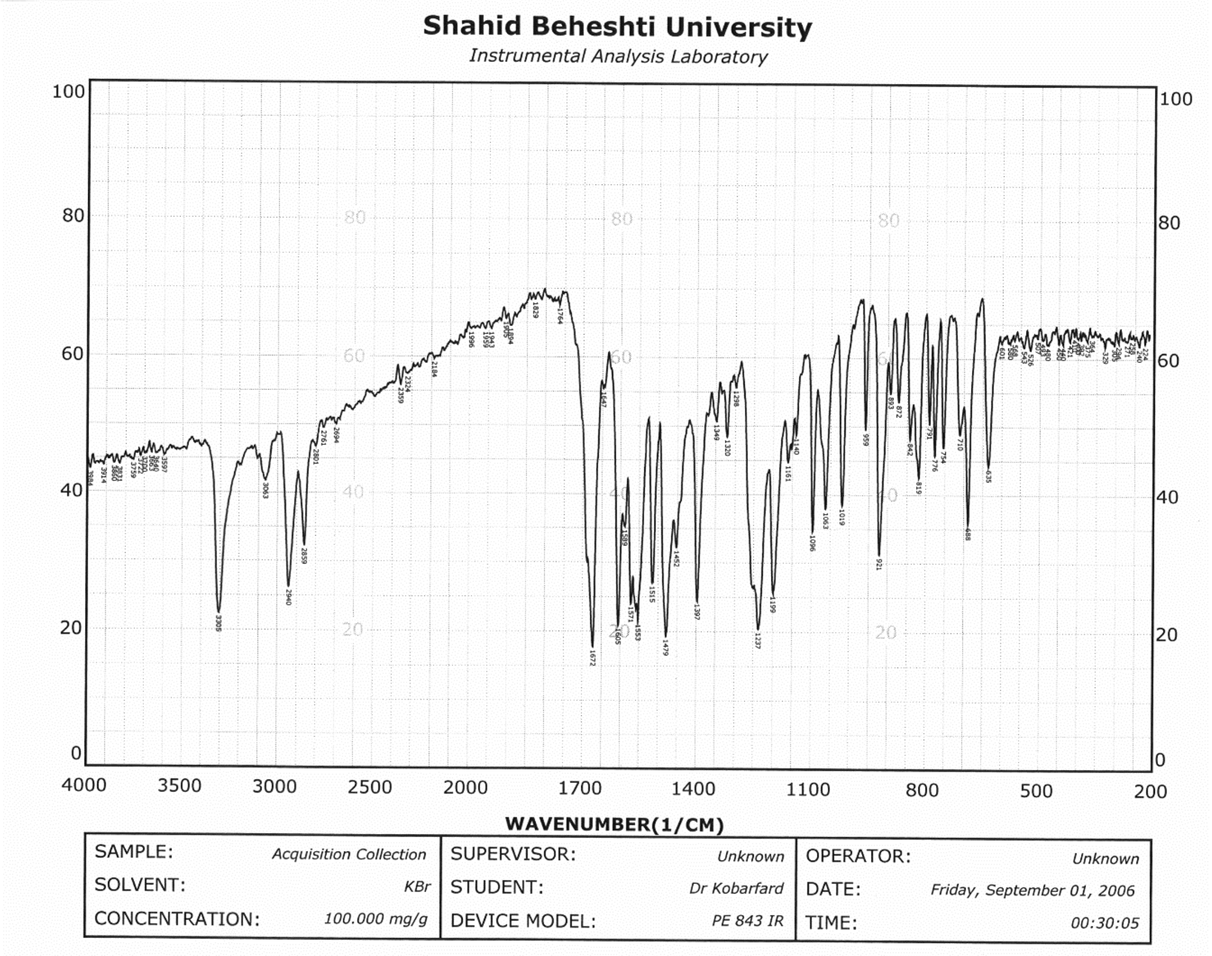

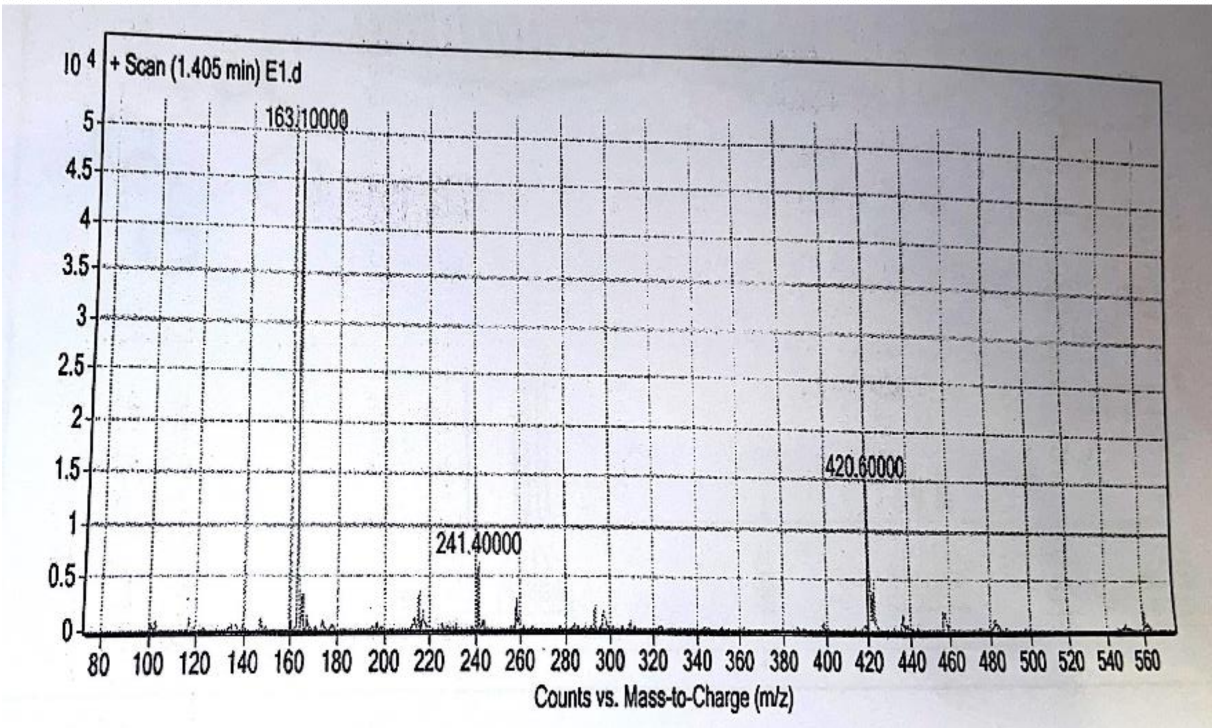

EXCLI Journal 2023;22:250-262 – ISSN 1611-2156
Received: November 28, 2022, accepted: February 02, 2023, published: February 20, 2023

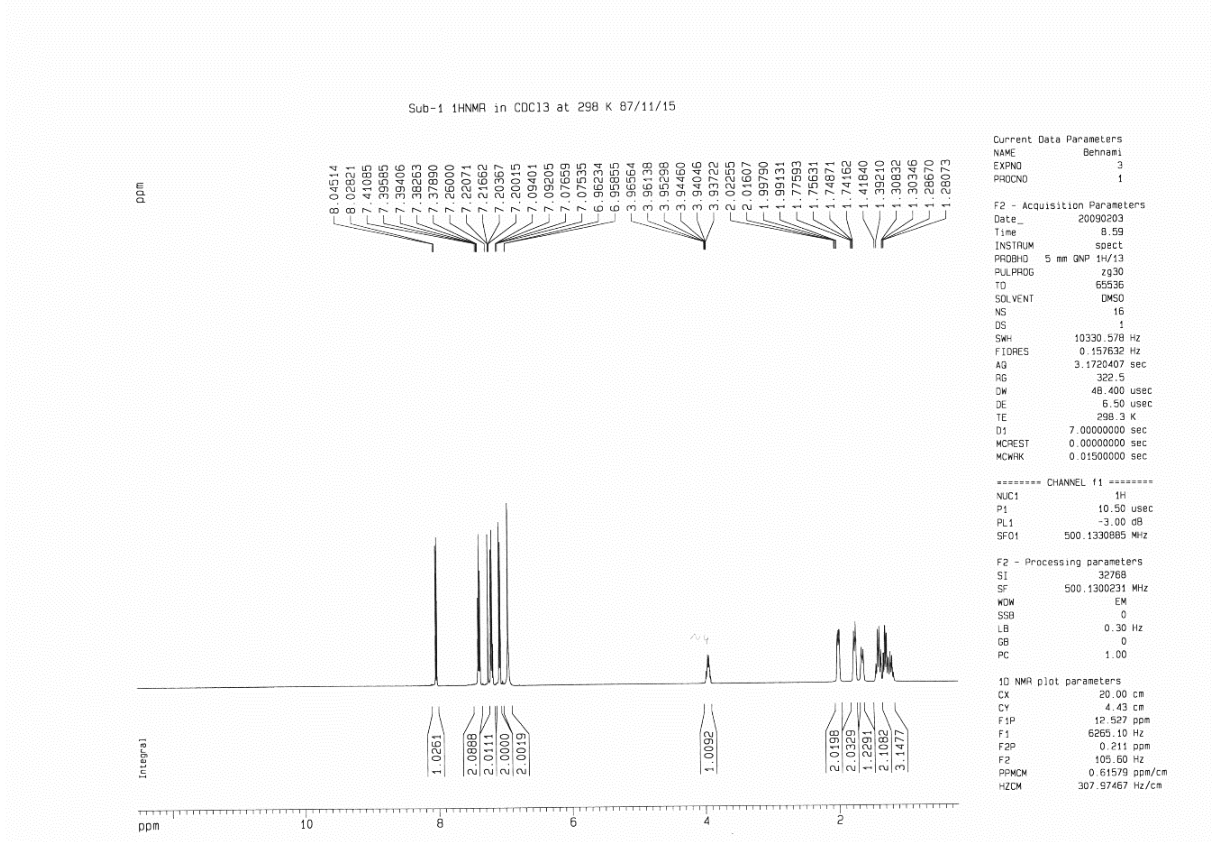

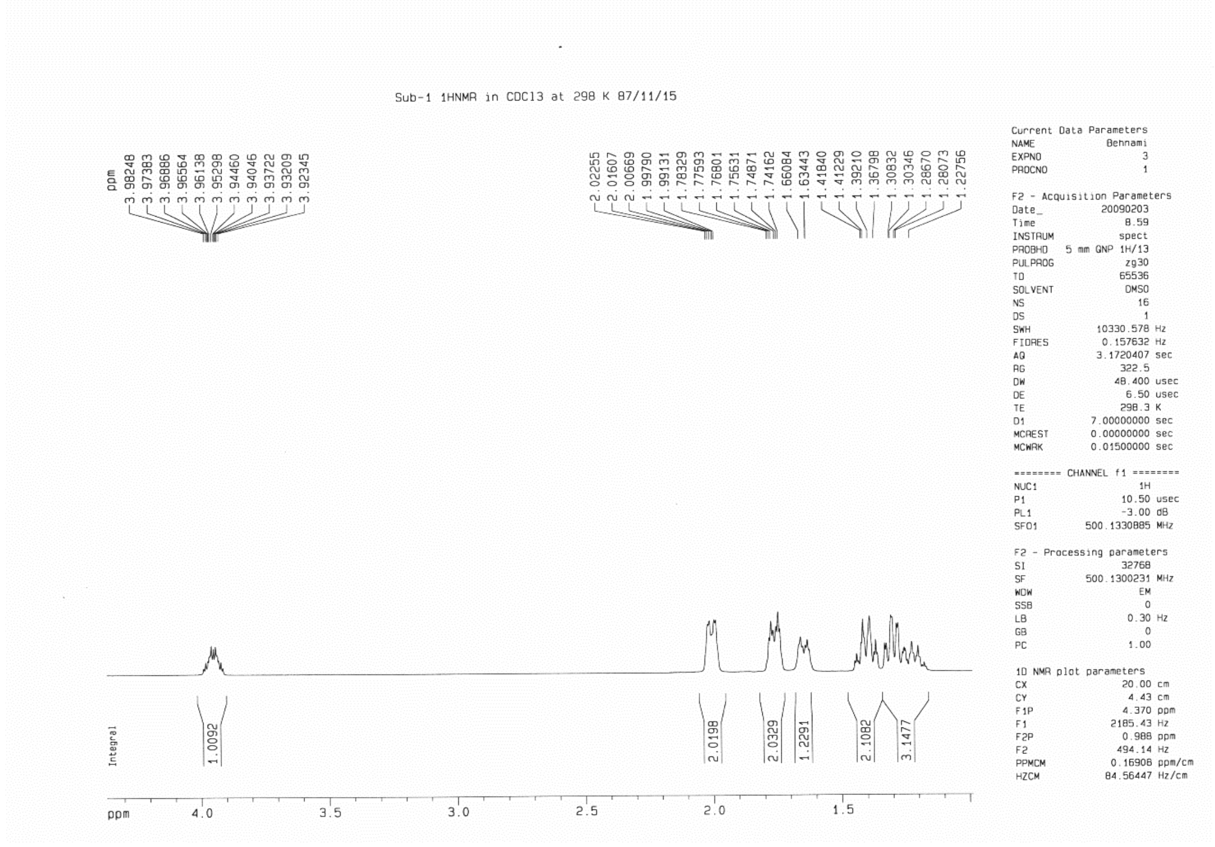

EXCLI Journal 2023;22:250-262 – ISSN 1611-2156
Received: November 28, 2022, accepted: February 02, 2023, published: February 20, 2023

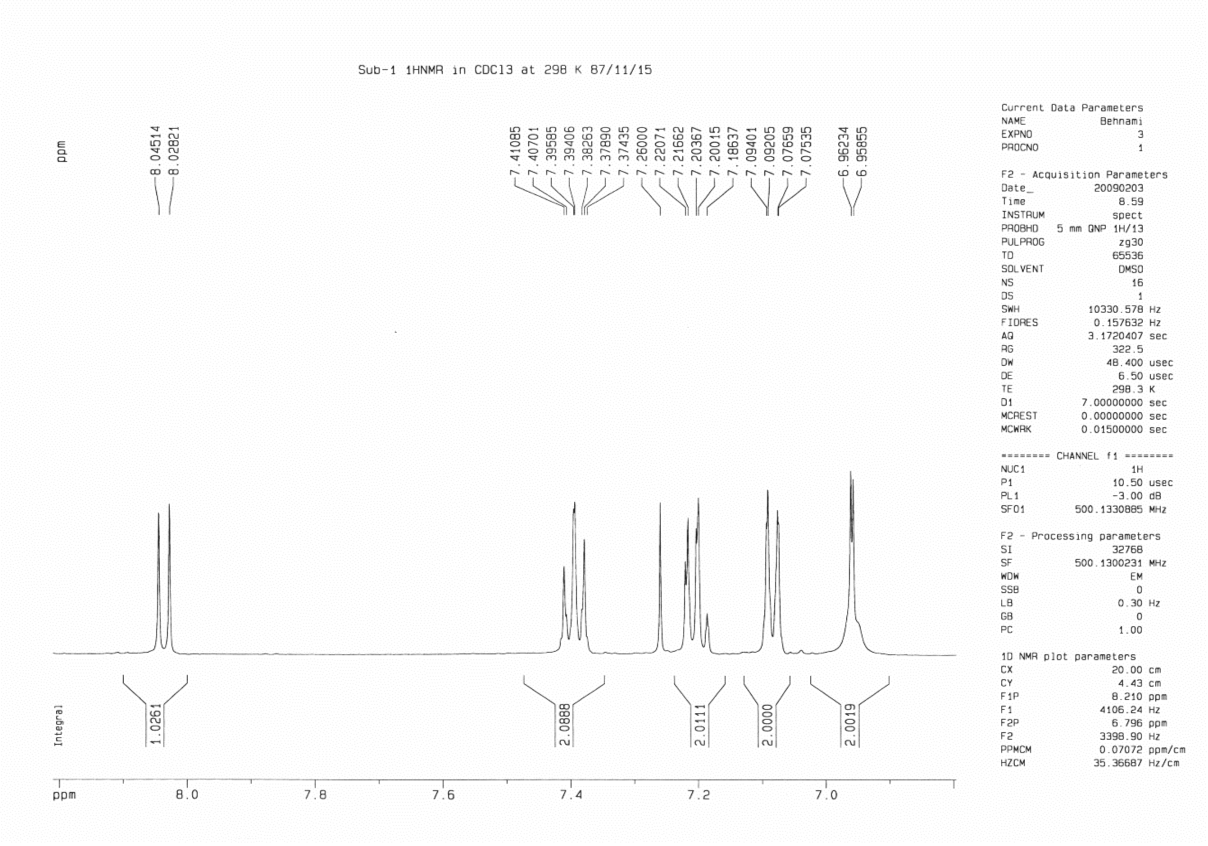

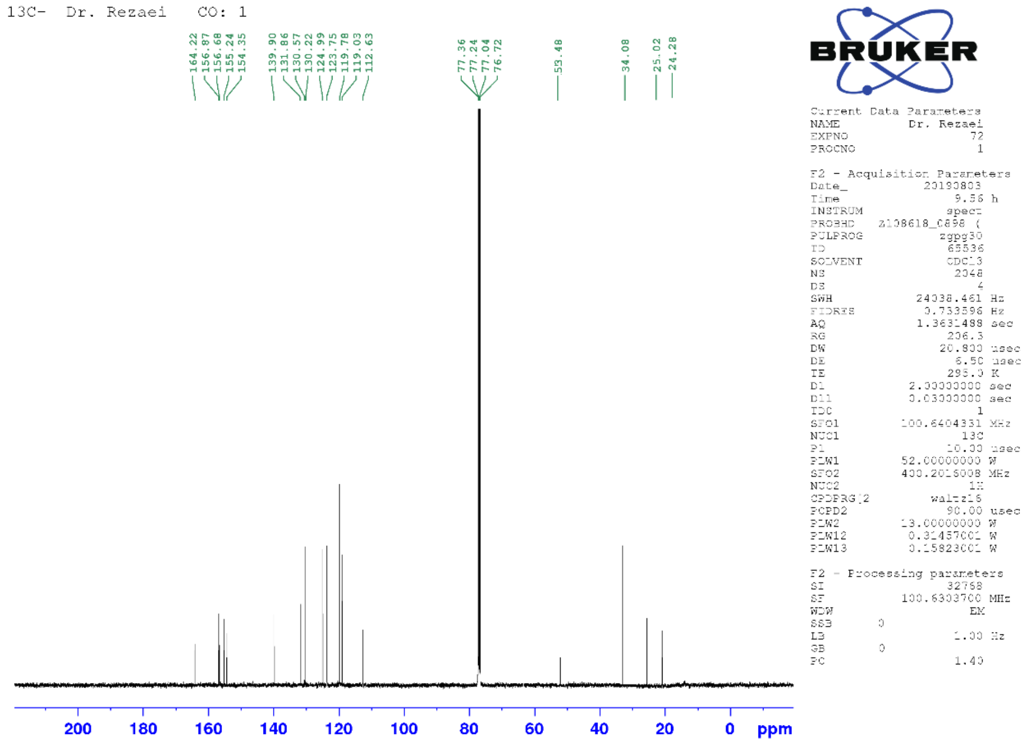

Received: November 28, 2022, accepted: February 02, 2023, published: February 20, 2023

Supplementary data (22) to Figure 3a: Raw data of latency of entrance to the dark compartment (second) in passive avoidance test. One-way ANOVA with Tukey's HSD post-hoc test was used (n=8)

No. of animal	Control	6a (1.25 mg/kg)	6a (2.5 mg/kg)	6a (5 mg/kg)	6a (10 mg/kg)	Diazepam (1 mg/kg)
1	320	320	420	480	320	262
2	480	380	400	440	460	260
3	360	393	389	344	407	299
4	480	467	376	343	230	240
5	420	480	480	380	400	320
6	480	480	400	407	420	200
7	420	420	480	480	480	206
8	420	420	363	251	326	333

Supplementary data (23) to Figure 3b: Raw data of Sleeping time (minute) in pentobarbital induced sleep test. One-way ANOVA with Tukey's HSD post-hoc test was used (n=8)

No. of ani-mal	Control	6a (1.25 mg/kg)	6a (2.5 mg/kg)	6a (5 mg/kg)	6a (10 mg/kg)	6a (10 mg/kg) + Flumazenil	Diazepam (2 mg/kg)
1	25	26	32	48	53	43	48
2	40	38	40	44	64	34	45
3	25	39	38	50	47	27	40
4	40	46	46	43	43	34	41
5	30	28	48	57	40	32	54
6	20	20	40	40	55	43	59
7	33	24	36	50	49	29	46
8	40	32	37	56	47	27	58

EXCLI Journal 2023;22:250-262 – ISSN 1611-2156
Received: November 28, 2022, accepted: February 02, 2023, published: February 20, 2023

Supplementary data (24) to Figure 4a: Raw data of % duration in open arms in elevated plus maze test. One-way ANOVA with Tukey's HSD post-hoc test was used (n=8)

No. of animal	Control	6a (1.25 mg/kg)	6a (2.5 mg/kg)	6a (5 mg/kg)	6a (10 mg/kg)	Diazepam (2 mg/kg)
1	43.46	35.00	38.62	51.49	57.55	94.26
2	0.00	33.00	39.93	55.45	53.71	85.00
3	44.35	44.00	49.52	55.59	52.34	90.00
4	53.91	40.00	43.28	56.51	56.82	66.15
5	46.00	47.00	37.18	61.83	62.42	54.00
6	0.00	36.21	50.82	52.34	54.62	60.00
7	31.41	35.00	33.27	51.49	58.96	68.00
8	34.06	41.00	36.78	48.34	73.22	57.00

Supplementary data (25) to Figure 4b: Raw data of % duration in close arms in elevated plus maze test. One-way ANOVA with Tukey's HSD post-hoc test was used (n=8)

No. of animal	Control	6a (1.25 mg/kg)	6a (2.5 mg/kg)	6a (5 mg/kg)	6a (10 mg/kg)	Diazepam (2 mg/kg)
1	56.53	65.00	61.37	48.50	42.44	5.73
2	100.00	67.00	60.06	44.54	46.28	15.00
3	55.64	56.00	50.48	44.40	47.65	10.00
4	46.08	60.00	56.72	43.48	43.17	33.84
5	53.98	53.00	62.82	38.16	37.57	46.00
6	100.00	64.00	49.18	47.65	45.37	40.00
7	68.58	65.00	66.73	48.50	41.04	32.00
8	65.93	59.00	63.22	51.65	26.78	43.00
